# Synthesis and Acid-Responsiveness of an Insulated π-Conjugated Polymer Containing Spiropyrans in Its Backbone

**DOI:** 10.3390/molecules24071301

**Published:** 2019-04-03

**Authors:** Hiromichi V. Miyagishi, Takashi Tamaki, Hiroshi Masai, Jun Terao

**Affiliations:** Department of Basic Science, Graduate School of Arts and Sciences, The University of Tokyo, 3-8-1 Komaba, Meguro-ku, Tokyo 153-8902, Japan; hero-miyagishi28@g.ecc.u-tokyo.ac.jp (H.V.M.); ctamaki@g.ecc.u-tokyo.ac.jp (T.T.); cmasai.h@mail.ecc.u-tokyo.ac.jp (H.M.)

**Keywords:** stimulus-responsive copolymer, sensors, spiropyran, acidochromism, supramolecular polymer, poly(*p*-phenylene ethynylene), permethylated α-cyclodextrins

## Abstract

A π-conjugated polymer containing spiropyrans (SPs), which could be almost completely converted to protonated merocyanines (MCH^+^) and back to the SP form by adding an acid and a base, respectively, was developed. The insulation of the π-conjugated polymer, referred to as insulated spiropyran-containing poly(*p*-phenylene ethynylene) (*ins*-SP-PPE), using permethylated α-cyclodextrins (PM α-CD) suppressed the π-π interaction between the polymer chains containing MCH^+^, and the installation of PM α-CD improved the switching ability of SPs. The polymer exhibited repeatable acidochromism with almost complete conversion between the SP and MCH^+^ forms. Photoluminescence measurements were conducted and the acid-induced luminescence quenching of the polymer in the solution was observed, which stemmed from energy transfer from the PPE to MCH^+^ moieties. In the solid state, the quantum yield of *ins*-SP-PPE was more than twice that of the uninsulated polymer, which derived from the insulation effects. The acid-induced luminescence quenching was also observed in the solid state.

## 1. Introduction

**Scheme 1:**
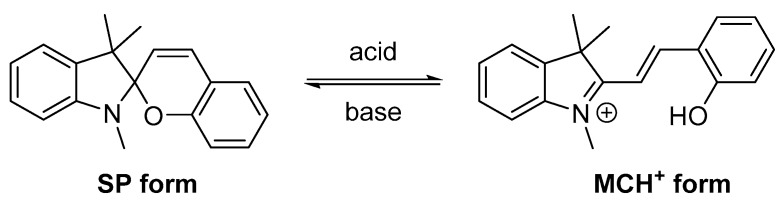
Acid- and base-induced conversion between spiropyrans (SP) and protonated merocyanines (MCH^+^).

Spiropyrans (SPs), first reported by Fischer and Hirshberg in 1952 [1], have been of great interest due to their ability to switch forms in response to various external stimuli, such as light, mechanical force and acid [2,3]. For example, irradiation of an SP with UV light causes isomerization of the SP to a merocyanine (MC), involving changes in the π-conjugated system, via heterolytic C-O bond cleavage. Under acid stimuli, SPs are converted to protonated merocyanines (MCH^+^) (Scheme 1) [4]. Because of the color differences between SPs and MCs (MCH^+^), SPs have been widely applied in electronic devices [5,6], bioimaging [7], and chemical sensors [8].

The incorporation of SPs into the backbones of π-conjugated polymers to allow the switching of their optical and electronic properties through the conversion of the SP moieties to MC moieties under external stimuli has already been reported. Ng et al. first described the incorporation of an SP into a poly(*p*-phenylene ethynylene) (polyPPE) backbone in 2006 [9] and demonstrated that the SP moieties in the backbone were incompletely isomerized to MC moieties under UV irradiation. Incorporation of SPs into poly(*p*-phenylene) [10], poly(*m*,*m*,*p*-phenylene) [11] and polyfluorene [12] has also been reported and the responses of the polymers to UV irradiation or mechanical force have been discussed. However, the isomerization of SPs in conjugated polymers showed low conversion and/or limited reversibility. As for UV light stimuli, one report indicated that the conversion of SPs in poly(*p*-phenylene) was approximately 16% and the SPs exhibited irreversible isomerization [10]. As for mechanical stimuli, one report indicated that the highest conversion of SPs was 44% and precipitation was observed after the proportion of MC exceeded the amount, which might have been caused by the π-π interactions between the MC moieties causing the polymer chains to have low solubility [12]. While several limitations of the isomerization have been demonstrated in conjugated SP polymers, the isomerization behavior of SPs under acid stimuli has not been studied for conjugated polymers [13].

Our group has developed a method to suppress the π-π interactions between π-conjugated polymer chains using covalently linked permethylated α-cyclodextrins (PM α-CDs), which three-dimensionally insulate the π-conjugated main chains [14,15]. The insulation using PM α-CDs gave the π-conjugated polymers high solubility in organic solvents and high solid-state emission quantum yields because the insulation prevented the π-π interaction between the polymer chains [16,17]. We have incorporated external-stimuli-responsive moieties into various π-conjugated polymers to prepare materials with changeable optical properties [14,18]. Herein, we report the synthesis of an insulated polyPPE containing SP moieties in its backbone (*ins*-SP-PPE). The conversion behavior of the SPs in *ins*-SP-PPE and the change in the optical properties of *ins*-SP-PPE were also investigated under acid stimulus.

## 2. Results and Discussion

### 2.1. Synthesis of Ins-SP-PPE and Unins-SP-PPE

**Scheme 2:**
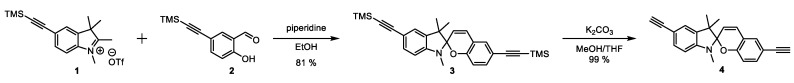

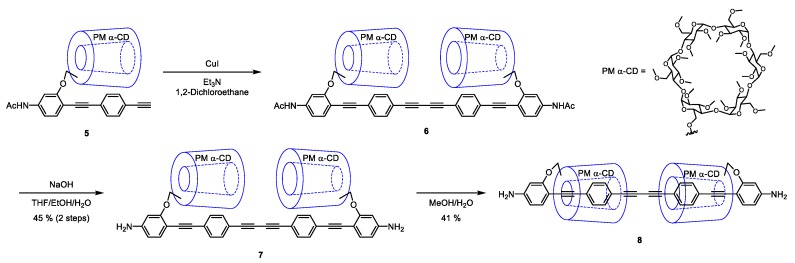
Synthesis of SP monomer **4** and insulated (*p*-phenylene ethynylene) (PPE) monomer **8**.

**Scheme 3:**
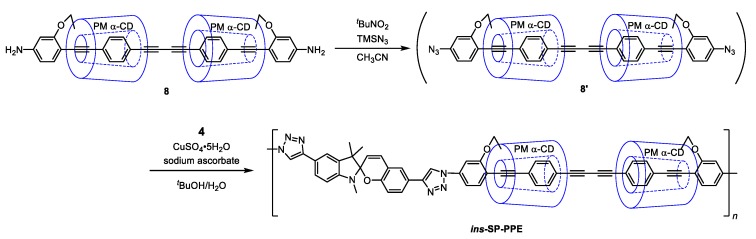
Polymerization of insulated spiropyran-containing poly(*p*-phenylene ethynylene) (*ins*-SP-PPE) using Huisgen 1,3-dipolar cycloaddition.

**Scheme 4:**
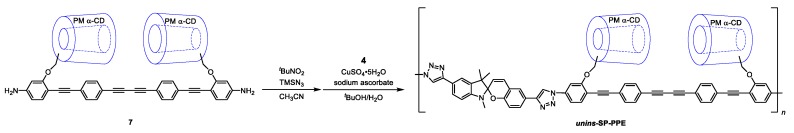
Polymerization of uninsulated (*unins*)-SP-PPE using Huisgen 1,3-dipolar cycloaddition.

**Figure 1:**
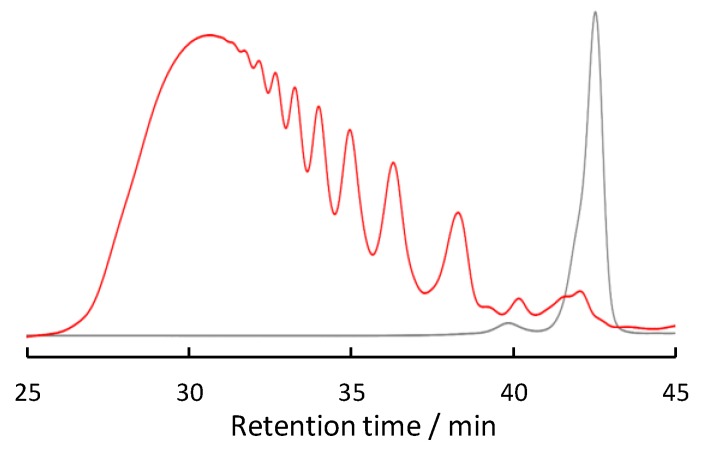
Gel-permeation chromatography (GPC) analysis of monomer **8** (gray line) and crude *ins*-SP-PPE (red line) after polymerization.

Copolymerization between SP monomer **4** and a derivative of PPE monomer **8** insulated with PM α-CDs was conducted via Cu-catalyzed Huisgen 1,3-dipolar cycloaddition to give *ins*-SP-PPE [19]. Indolenium salt **1** and a derivative of salicylic aldehyde **2** were heated in the presence of piperidine to give SP **3**, and subsequent deprotection of the trimethylsilyl group afforded SP monomer **4** (Scheme 2). Glaser coupling of the previously reported PPE **5** and subsequent hydrolysis of the acetamide gave uninsulated PPE monomer **7**. Insulated monomer **8** was synthesized by the self-inclusion of **7** [15,20]. The quantitative insulation was confirmed by the characteristic downfield shift of the signals of the aromatic protons on the insulated PPE (Figure S14). Monomer **8** was kinetically isolated even in nonpolar solvents because the activation energy of the dethreading of **8** was high enough to prevent it from occurring [20]. Monomer **8** was copolymerized with **4** after the conversion of the amine groups on **8** to azide groups (Scheme 3). As shown in Figure 1, the gel-permeation chromatography (GPC) chart evidenced the formation of *ins*-SP-PPE with the consumption of the insulated PPE monomer **8**. In order to remove oligomers, the crude *ins*-SP-PPE was treated with preparative GPC to give purified *ins*-SP-PPE (*M*_w_ = 1.3 × 10^5^, *M*_n_ = 5.1 × 10^4^). In addition, uninsulated PPE polymer *unins*-SP-PPE (*M*_w_ = 4.2 × 10^4^, *M*_n_ = 3.0 × 10^4^) was also synthesized from monomer **7** under the same conditions as those for the synthesis from **8** (Scheme 4). The lower *M*_w_ and *M*_n_ probably stemmed from the low solubility of uninsulated conjugated polymers caused by the π-π stacking between the polymer chains.

### 2.2. UV-Vis Titration with Acid and Base Stimuli

**Figure 2:**
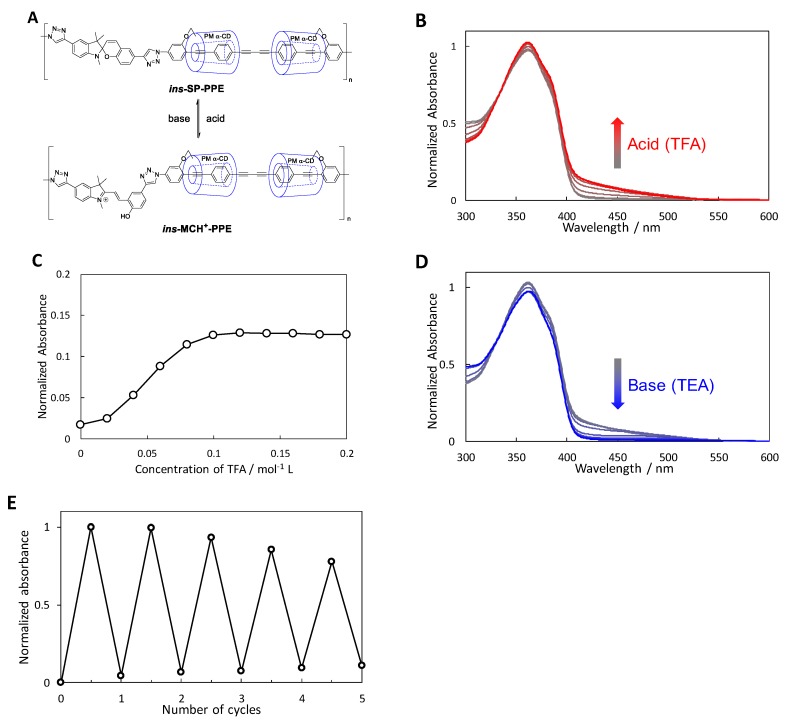
(**A**) Acid- and base-induced structural conversion between *ins*-SP-PPE and *ins*-MCH^+^-PPE. (**B**) Ultraviolet-visible (UV-Vis) spectroscopy spectra of *ins*-SP-PPE (2.0 × 10^−5^ M in CH_2_Cl_2_) upon addition of different amounts of trifluoroacetic acid (TFA) (0–0.2 M). (**C**) A change of normalized absorbance of *ins*-SP-PPE at 420 nm under the addition of TFA (0–0.2 M). (**D**) UV-Vis spectra of *ins*-SP-PPE (2.0 × 10^−5^ M in CH_2_Cl_2_) upon addition of different amounts of triethylamine (TEA) (0–0.2 M) following the TFA addition (0.2 M). (**E**) Repeatable switching of the absorbance of *ins*-SP-PPE (2.0 × 10^−5^ M in CH_2_Cl_2_) at 420 nm by the addition of TFA and TEA.

Ultraviolet-visible (UV-Vis) spectroscopy titration was conducted to investigate the acidochromic properties of *ins*-SP-PPE and *unins*-SP-PPE. The acid stimuli were used because one report suggested that the acid stimuli were able to quantitatively convert SPs to MCH^+^ in the polymer backbone [13]. It was expected that *ins*-SP-PPE would be converted to *ins*-MCH^+^-PPE by an acid stimulus (Figure 2A). The UV-Vis spectrum of *ins*-SP-PPE in CH_2_Cl_2_ (2.0 × 10^−5^ M) contained an absorbance peak at 362 nm, which was attributed to PPE (Figure 2B), and the addition of trifluoroacetic acid (TFA) to the solution led to the appearance of a broad absorption at greater than 420 nm, which was attributed to MCH^+^ [4], with an isosbestic point at 333 nm. Additionally, the almost complete conversion of SP moieties in *ins*-SP-PPE was confirmed because the absorbance at 420 nm was saturated under the addition of more than 0.1 M TFA (Figure 2C). The formation of precipitation could be denied because any decrease of absorbance was not observed. Subsequently, to confirm the reversibility of this acid-induced conversion, triethylamine (TEA) was added into the solution as a base (Figure 2D), which resulted in the decrease of the absorption at greater than 420 nm, indicating that the conversion from the MCH^+^ form to the SP form occurred. The almost complete conversion of MCH^+^ to SP was also confirmed by the saturation of the absorbance. Similar acidochromic behaviors were observed in *unins*-SP-PPE (Figure S27), which probably derived from the high solubility of bulky side chains of PM α-CD in *unins*-SP-PPE or the low degree of polymerization of *unins*-SP-PPE. These results confirmed the acidochromism between *ins*-SP-PPE and *ins*-MCH^+^-PPE. This acidochromic behavior was repeatedly observed upon the addition of acid and base (Figure 2E).

### 2.3. ^1^H-NMR Study

**Figure 3:**
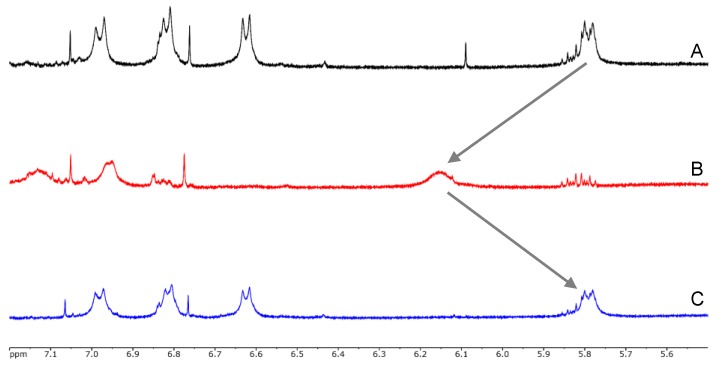
A proton nuclear magnetic resonance (^1^H-NMR) spectra (aromatic region) of *ins*-SP-PPE before (**A**, black line) and after adding TFA (**B**, 0.04 M, red line) and then TEA (**C**, 0.08 M, blue line). Gray allows indicate the shift of signals deriving from vinyl protons of SP and MCH^+^.

A proton nuclear magnetic resonance (^1^H NMR) study was conducted to investigate the acidochromic behavior of *ins*-SP-PPE. As shown in Figure 3A, the SP signals were observed at 6.98, 6.84, 6.62, and 5.79 ppm. The addition of TFA resulted in the downfield shift of these signals as clearly shown by a vinyl proton of SP moieties (Figure 3B). This shift indicated the conversion of SP moieties to MCH^+^ ones, not to any other species because the similar shift was observed in the unsubstituted SP, 1′,3′,3′-trimethylspiro(2*H*-1-benzopyran-2,2′-indoline) (Figure S20). Similar phenomena were observed in *unins*-SP-PPE (Figure S23), which is consistent with the results of UV-Vis titration. The shifted signals of SP moieties in *ins*-SP-PPE was reset to the original position by adding an excess amount of TEA (Figure 3C), which confirmed the reversible conversion between SP and MCH^+^ moieties.

### 2.4. Photoluminescence Study in the Solution and Solid State

**Figure 4:**
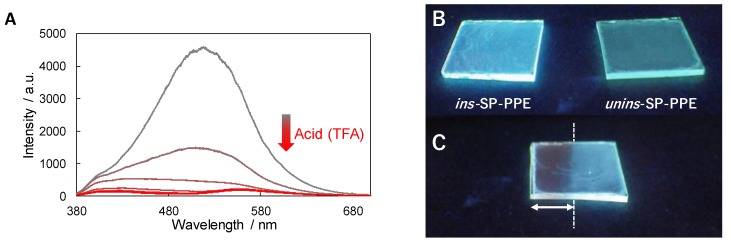
(**A**) Emission spectra of *ins*-SP-PPE upon adding different amounts of TFA (0–0.2 M). The solution of *ins*-SP-PPE was excited at 362 nm. Photographs under UV irradiation at 365 nm of (**B**) *ins*-SP-PPE and *unins*-SP-PPE films on a glass substrate and (**C**) fluorescence quenching of *ins*-SP-PPE before (right side) and after (left side) dipping in aqueous 0.1 M HCl for 10 s. The double-headed arrow marks the dipped region of the film.

Photoluminescence measurements were conducted to investigate the effects of the acid-induced SP conversion. A peak around 520 nm was observed in the emission spectrum of *ins*-SP-PPE in CH_2_Cl_2_ solution (2.0 × 10^−5^ M) (Figure 4A), which stemmed from the PPE chains. After the addition of TFA to the solution, significant quenching of the fluorescence was observed. A similar phenomenon was previously reported by Jung et al. [8], which was attributed to fluorescence quenching of polythiophene via energy transfer between polythiophene and MC moieties. Accordingly, the quenching of *ins*-SP-PPE was probably caused by energy transfer from the PPE moieties to the MCH^+^ moieties.

The luminescence properties of the polymers and the acid responsiveness of *ins*-SP-PPE in the solid state were also examined. Films of *ins*-SP-PPE and *unins*-SP-PPE were prepared on glass substrates by drop-casting their solutions. Under irradiation with UV light (365 nm), the film of *ins*-SP-PPE exhibited stronger emission than that of *unins*-SP-PPE (Figure 4B). The quantum yields of *ins*-SP-PPE and *unins*-SP-PPE in the solid state were 5.4% and 2.4%, respectively. The fact that the quantum yield of the insulated polymer was more than twice that of the uninsulated one was attributed to the suppression of the π-π interaction between the conjugated polymers. Furthermore, dipping the *ins*-SP-PPE film into 0.1 M HCl aqueous solution for 10 s resulted in the quenching of luminescence only in the dipped region (Figure 4C). As mentioned above, this was because of energy transfer between the π-conjugated backbone and MCH^+^ moieties.

## 3. Materials and Methods

The general remarks, the synthetic procedures of monomer **4**, **8** and *unins*-SP-PPE, and the preparation methods of the films of *ins*-SP-PPE and *unins*-SP-PPE are available in the Supplementary Materials.

### Synthesis of Ins-SP-PPE

To a solution of PPE monomer **8** (45 mg, 16 μmol, 1.0 eq.) in dehydrated acetonitrile (1.6 mL), *tert*-butyl nitrite (37 μL, 0.31 mmol, 20 eq.) and trimethylsilyl azide (62 μL, 0.47 mmol, 30 eq.) were added at 0 °C and then stirred for 2 h at room temperature. After the reaction, the solvent was evaporated in vacuum. The residue was diluted with chloroform and H_2_O and extracted with chloroform and diethyl ether. The combined organic layer was dried over MgSO_4_, filtrated, and evaporated in vacuum to afford the product as a yellow solid. Without further purification, the mixture of the solid, **4** (5.3 mg, 16 μmol, 1.0 eq.), sodium ascorbate (19 mg, 94 μmol, 6.0 eq.), and CuSO_4_·5H_2_O (24 mg, 94 μmol, 6.0 eq.) in H_2_O (391 μL) and *^t^*BuOH (391 μL) was stirred overnight at room temperature. After the reaction, the mixture was diluted with chloroform and saturated aqueous NH_4_Cl solution and extracted with chloroform. The combined layer was washed with saturated aqueous NaHCO_3_, dried over MgSO_4_, filtrated, and evaporated in vacuum. To remove the oligomers, the residue was purified by preparative GPC with chloroform as the eluent to afford product *ins*-SP-PPE as a yellow solid (22 mg, 43%, *M*_w_ = 1.3 × 10^5^, *M*_n_ = 5.1 × 10^4^, PDI = 2.5); ^1^H-NMR (500 MHz; CDCl_3_): δ = 8.18–8.08 (m, 6H, triazole–H, ArH), 7.74–7.58 (m, 14H, ArH), 6.98 (d, *J* = 10.0 Hz, 1H, –CH=CH–), 6.82 (d, *J* = 7.8 Hz, 1H, ArH), 6.63 (d, *J* = 8.3 Hz, 1H, ArH), 5.79 (d, *J* = 9.6 Hz, 1H, –CH=CH–), 5.13–2.97 (m, 186H, CD–H, OCH_3_), 2.82 (s, 3H, N–CH_3_), 1.40 (s, 3H, *gem*–CH_3_), 1.25 (s, 3H, *gem*–CH_3_).

## 4. Conclusions

An insulated π-conjugated polymer, *ins*-SP-PPE, was synthesized by copolymerization of SP monomer **4** and insulated PPE monomer **8** via Cu-catalyzed Huisgen 1,3-dipolar cycloaddition. UV-Vis titrations revealed that the polymer exhibited repeatable acidochromism and the SP moieties in the polymer were almost completely converted to MCH^+^ ones. ^1^H-NMR study supported the fact that the SP moieties in the polymer converted to MCH^+^ moieties, not to any other species. In addition, the acid-induced luminescence quenching of *ins*-SP-PPE was observed in the solution and solid state. We will further investigate the chromic properties of *ins*-SP-PPE induced by other stimuli.

## Supplementary Materials

Supplementary Materials, including general remarks, synthetic procedures, NMR spectra, charts of GPC, UV-Vis spectra, emission spectra, and a photograph of a polymer film, are available online.
