# Supplementary material for: Synthesis and Acid-Responsiveness of an Insulated π-Conjugated Polymer Containing Spiropyrans in Its Backbone
Source: Molecules. 2019 Apr 3;24(7):1301. doi: 10.3390/molecules24071301 (PMC6480293; doi:10.3390/molecules24071301)
Supplement: Supplementary file 1 [file molecules-24-01301-s001.pdf]

Supporting Information

Synthesis and Acid-Responsiveness of an Insulated π-Conjugated Polymer Containing Spiropyrans in Its Backbone

Hiromichi V. Miyagishi, Takashi Tamaki, Hiroshi Masai, Jun Terao *

Department of Basic Science, Graduate School of Arts and Sciences, The University of Tokyo, 3-8-1 Komaba, Meguro-ku, Tokyo 153-8902, Japan.

*Correspondence: cterao@mail.ecc.u-tokyo.ac.jp

Table of Contents

1. General Remarks 3
1.1 Materials 3
1.2 Experimental equipment and procedures 3
2. Synthetic procedures 4
2.1 Synthesis of compound **1** 4
2.2 Synthesis of compound **2** 5
2.3 Synthesis of SP monomer **4** 5
2.4 Synthesis of PPE monomers **7** and **8** 6
2.5 Synthesis of ***unins*-SP-PPE**. 8
2.6 Preparation of films of ***ins*-SP-PPE** and ***unins*-SP-PPE** 8
3. NMR spectra 9
3.1 NMR spectra of **S1** 9
3.2 NMR spectra of **S2** 10
3.3 NMR spectrum of **2** 11
3.4 NMR spectra of **3** 12
3.5 NMR spectra of **4** 13
3.6 NMR spectra of **7** 14
3.7 NMR spectra of **8** 15
3.8 NMR spectra of 7 and 8 (aromatic region) 16
3.9 NMR spectra of ***ins*-SP-PPE** and ***unins*-SP-PPE** 17
3.10 NMR titration 18
4. GPC charts 22
5. UV-Vis spectra 24
6. Photoluminescence study 24
5. References 25

1. General Remarks

1.1 Materials

Unless otherwise noted, manipulations were performed under nitrogen atmosphere using standard Schlenk-type glassware in a dual-manifold Schlenk line. Unless otherwise stated, commercially available chemicals were used as received. Reaction solvents were degassed by nitrogen bubbling, before using. Compound **5** [1] and unsubstituted SP [2] was prepared according to the previously reported procedure.

1.2 Experimental equipment and procedures

NMR spectroscopy

^{1}H NMR (500 MHz) and ^{13}C {^{1}H} NMR (126 MHz) spectra were measured with a Bruker AVANCE-500 spectrometer. The ^{1}H-NMR chemical shifts are reported relative to tetramethylsilane (TMS) (0.00 ppm). The ^{13}C-NMR chemical shifts are reported relative to TMS (0.00 ppm) or deuterated solvents (77.16 ppm for $CDCl_3$).

High-Resolution Mass Spectroscopy (HR-MS)

Electrospray ionization time-of-flight (ESI-TOF) mass spectra were obtained using a Waters Xev G2-S Tof mass spectrometer.

Analytical gel permeation chromatography (Analytical GPC)

Analytical GPC was performed with a GL-Science GL-7400 HPLC System equipped with Shodex KF-802, -802.5, -803columns, a GL-7410 HPLC pump, a GL-7400 UV detector, and a GL-7454 RI detector using THF as eluent at a flow rate of 0.6 mL min^{-1}. Average molecular weights of polymers were estimated with polystyrenes as calibration standards.

Preparative recycling gel permeation chromatography (Preparetive GPC)

Preparative recycling GPC was performed with a SHIMADZU LC-20AP System equipped with a Shodex K-4002L or K-4003L column, a SHIMADZU SPD-20A, and a SHIMADZU RID-10A, using $CHCl_3$ as the eluent at a flow rate of 14 mL min^{-1}.

UV-Vis spectroscopy

UV-Vis spectra were measured with a SHIMADZU UV-2600 spectrophotometer using CH_2Cl_2 as the solvent. For UV-Vis titration, trifluoroacetic acid (TFA, 4.6 μL × 10) or triethylamine (TEA, 8.5 μL × 10) was added into the solution of samples (2.0×10^{-5} M, 3 mL). For repeated test of the acidochromism, small excess amounts of TFA and TEA were added into the solution of samples (2.0×10^{-5} M, 3 mL). The amounts of TFA and TEA were summarized below. 1st cycle: TFA 46 μL and TEA 40 μL. 2nd cycle: TFA 51 μL and TEA 44 μL. 3rd cycle: 56 μL and 48 μL. 4th cycle: 62 μL and 53 μL. 5th cycle: 68 μL and 59 μL.

Photoluminescence spectroscopy

Fluorescence spectra were measured with a HITACHI F-7000 model equipped with a 150 W Xenon lamp using CH_2Cl_2 as the solvent. Titration experiments were conducted with the same method as the UV-Vis titration.

Quantum Yield measurement

Absolute quantum yields were determined by a calibrated integrating sphere system (Hamamatsu C11347). This system consists of an excitation light source, a sample holder mounted in an integrating sphere and a multi-channel CCD spectrometer.

2. Synthetic procedures

2.1 Synthesis of compound **1**

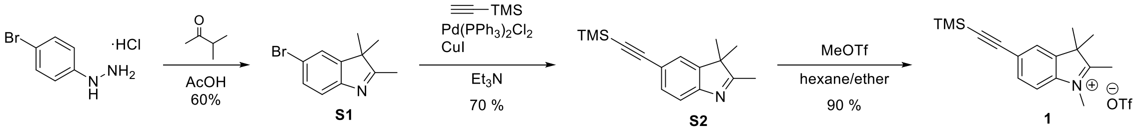

Scheme S1. Synthesis of compound **1**.

Compound S1. To a solution of 4-bromophenylhydrazine hydrochloride (1.00 g, 4.28 mmol, 1.0 eq.) in acetic acid (14 mL), 3-methylbutan-2-one (0.692 mL, 6.42 mmol, 1.5 eq.) was added. The mixture was heated under reflux for 21 h. After the mixture cooled to room temperature, the solvent was removed in vacuum. The residue was diluted with dichloromethane (40 mL) and saturated $NaHCO_3$ aq., and extracted with dichloromethane (2 × 40 mL). The organic layers were combined, washed with brine, dried over Na_2SO_4, filtered, and evaporated in vacuum. The residue was purified by flash column chromatography eluting with hexane/EtOAc (3:2) to afford product **S1** as brown oil (614 mg, 60%); ^{1}H-NMR (500 MHz; $CDCl_3$): δ = 7.43-7.38 (m, 3H), 2.26 (s, 3H), 1.30 (s, 6H). ^{13}C-NMR (126 MHz; $CDCl_3$): δ = 188.4, 152.7, 147.8, 130.6, 124.8, 121.3, 118.8, 54.1, 22.9, 15.4. The ^{1}H-NMR and ^{13}C-NMR spectra were consistent with those previously reported [3].

Compound S2. This compound was synthesized according to the previously published procedures [4]; ^{1}H-NMR (500 MHz; $CDCl_3$): δ = 7.45-7.42 (m, 2H), 7.39 (s, 1H), 2.28 (s, 3H), 1.29 (s, 6H), 0.26 (s, 9H). ^{13}C-NMR (126 MHz; $CDCl_3$): δ = 189.6, 154.1, 145.8, 132.1, 125.2, 119.88, 119.75, 105.7, 93.9, 53.9, 23.1, 15.7, 0.2. The ^{1}H-NMR and ^{13}C-NMR spectra were consistent with those previously reported [4].

Compound 1. To a solution of **S2** (250 mg, 1.02 mmol, 1.0 eq.) in dehydrated hexane (1.3 mL) and diethyl ether (1.9 mL), methyl trifluoromethanesulfonate (134.1 μL, 1.22 mmol, 1.2 eq.) was added. Instantly, a white precipitate formed. The solid was filtered off and washed with cold diethyl ether. The solid was dried in vacuum to afford product **1** as a white solid (369 mg). This compound was used in the next reaction without further purification.

2.2 Synthesis of compound **2**

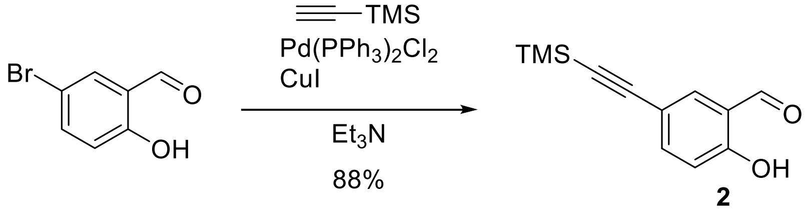

Scheme S2. Synthesis of compound **2**.

Compound 2. This compound was synthesized according to the previously published procedures [5]; ^{1}H-NMR (500 MHz; $CDCl_3$): δ = 11.10 (s, 1H), 9.85 (s, 1H), 7.70 (d, J = 1.9 Hz, 1H), 7.60 (dd, J = 8.6, 2.1 Hz, 1H), 6.93 (d, J = 8.7 Hz, 1H), 0.25 (s, 9H). The ^{1}H-NMR data were consistent with that previously reported [5].

2.3 Synthesis of SP monomer **4**

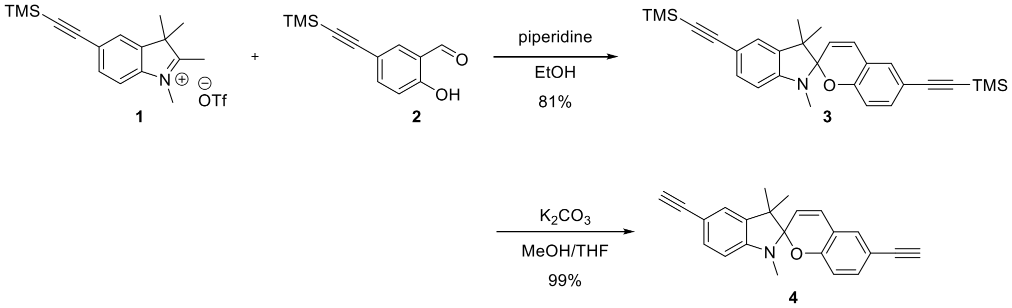

Scheme S3. Synthesis of SP monomer **4**.

Compound 3. To a solution of **1** (369 mg, 0.879 mmol, 1.0 eq.) and **2** (192 mg, 0.879 mmol, 1.0 eq.) in ethanol (8.8 mL), piperidine (95.7 μL, 0.967 mmol, 1.1 eq.) was added and then heated under reflux for 4 h. After the mixture cooled to room temperature, the solvent was evaporated in vacuum. The residue was filtrated through SiO_2 gel with hexane/EtOAc (19:1) and the eluent was evaporated in vacuum. The residue was recrystallized from EtOH to afford product **3** as a white solid (335 mg, 81%); ^{1}H-NMR

(500 MHz; $CDCl_3$): δ = 7.32 (dd, J = 8.0, 1.5 Hz, 1H, ArH), 7.21 (dd, J = 8.3, 2.0 Hz, 1H, ArH), 7.19 (d, J = 1.8 Hz, 1H, ArH), 7.16 (d, J = 1.4 Hz, 1H, ArH), 6.80 (d, J = 10.3 Hz, 1H, –CH=CH–), 6.59 (d, J = 8.3 Hz, 1H, ArH), 6.41 (d, J = 8.0 Hz, 1H, ArH), 5.67 (d, J = 10.2 Hz, 1H, –CH=CH–), 2.70 (s, 3H, N–CH_3), 1.25 (s, 3H, *gem*–CH_3), 1.13 (s, 3H, *gem*–CH_3), 0.24 (s, 9H, $SiMe_3$), 0.23 (s, 9H, $SiMe_3$). ^{13}C-NMR (126 MHz; $CDCl_3$): δ = 154.7, 148.5, 136.8, 133.9, 132.6, 130.6, 129.2, 125.5, 119.7, 118.7, 115.2, 115.0, 113.3, 106.77, 106.62, 105.1, 104.7, 92.5, 91.3, 51.8, 28.9, 25.8, 20.1, 0.35, 0.21. HR-MS (ESI-TOF-MS) *m/z*: $[M+H]^+$ calcd for $C_{29}H_{36}NOSi_2$ 470.2336, found 470.2334.

Compound 4 A mixture of **3** (200 mg, 0.616 mmol, 1.00 eq) and K_2CO_3 (400 mg, 2.89 mmol, 4.69 eq.) in MeOH (4 mL) and THF (4 mL) was stirred for 3 h at room temperature. The suspension was evaporated in vacuum and purified by column chromatography eluting with hexane/EtOAc (9:1) to afford product as a white solid (137 mg, 99%); ^1H-NMR (500 MHz; $CDCl_3$): δ = 7.38 (dd, J = 8.0, 1.6 Hz, 1H, ArH), 7.26 (dd, J = 8.4, 2.0 Hz, 1H, ArH), 7.24 (d, J = 2.0 Hz, 1H, ArH), 7.21 (d, J = 1.4 Hz, 1H, ArH), 6.85 (d, J = 10.3 Hz, 1H, –CH=CH–), 6.67 (d, J = 8.4 Hz, 1H, ArH), 6.47 (d, J = 8.0 Hz, 1H, ArH), 5.72 (d, J = 10.2 Hz, 1H, –CH=CH–), 3.02 (s, 1H, –C≡CH), 3.00 (s, 1H, –C≡CH), 2.76 (s, 3H, N–CH_3), 1.30 (s, 3H, *gem*–CH_3), 1.18 (s, 3H, *gem*–CH_3). ^{13}C-NMR (126 MHz; $CDCl_3$): δ = 154.7, 148.5, 136.7, 133.8, 132.5, 130.6, 129.0, 125.5, 119.6, 118.6, 115.2, 113.7, 112.1, 106.5, 104.6, 84.9, 83.4, 75.6, 74.7, 51.7, 28.8, 25.7, 19.9. HR-MS (ESI-TOF-MS) *m/z*: $[M+H]^+$ calcd for $C_{23}H_{20}NO$ 326.1545, found 326.1545.

2.4 Synthesis of PPE monomers **7** and **8**

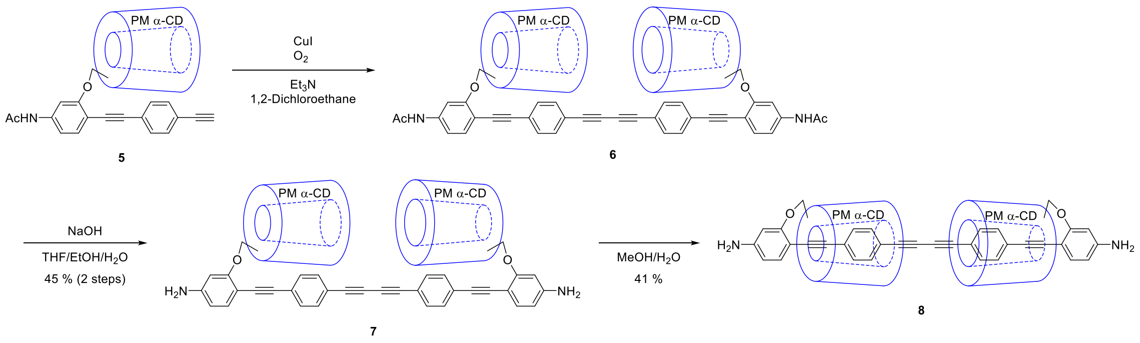

Scheme S4. Synthesis of PPE monomers **7** and **8**.

Compound 6. A mixture of **5** (300 mg, 0.204 mmol, 1.0 eq.) and CuI (1.25 g, 6.56 mmol, 32 eq.) in 1,2-dichloroethane (6 mL) and Et_3N (13 mL) was heated for 13 h at 60 °C under air. After the mixture cooled to room temperature, satulated NH_4Cl aq. (100 mL)

was added. The mixture was extracted with dichloromethane (2×50 mL) and then diethyl ether (50 mL). The combined organic layer was washed with saturated NH_4Cl aq., and the aqueous layer was extracted with dichloromethane and diethyl ether. The combined organic layer was dried over $MgSO_4$, filtrated, and evaporated in vacuum. The residue was purified by preparative GPC with chloroform as eluent to afford product **6** as a yellow solid (202 mg). This compound was used in the next reaction withous further purification.

Compound 7. 6 (202 mg, 68.8 μmol) was dissolved in THF (22.5 mL), EtOH (9 mL) and H_2O (9 mL). To the solution, NaOH (7.2 g) was added and heated for 3 days at 40 °C. After the mixture cooled to room temperature, Brine (50 mL), H_2O (30 mL), chloroform (100 mL) was added and extracted with chloroform (2×50 mL) and then diethyl ether (50 mL). The combined organic layer was dried over $MgSO_4$, filtrated, and evaporated in vaccum. The residue was purified by preparative GPC with chloroform as eluent to afford product **7** as a yellow solid (131 mg, 2 steps 45%); ^{1}H-NMR (500 MHz; $CDCl_3$): δ = 7.52 (d, J = 8.4 Hz, 4H, ArH), 7.45 (d, J = 8.4 Hz, 4H, ArH), 7.25 (d, J = 8.2 Hz, 2H, ArH), 6.24 (dd, J = 8.2, 1.7 Hz, 2H, ArH), 6.20 (d, J = 1.6 Hz, 2H, ArH), 5.16-3.05 (m, 190H, CD–H, OCH_3, NH_2). ^{13}C-NMR (126 MHz; $CDCl_3$): δ = 160.7, 148.6, 134.3, 132.3 (peaks overlapped), 131.2 (peaks overlapped), 125.3, 120.3, 107.5, 102.5, 100.5, 100.25, 100.18, 100.09 (peaks overlapped), 99.6, 99.2, 91.4, 90.2, 82.74, 82.61, 82.52 (peaks overlapped), 82.37, 82.33, 82.20 (peaks overlapped), 82.13, 82.05, 82.02, 81.23 (peaks overlapped), 81.17 (peaks overlapped), 81.11, 75.3, 71.74, 71.61, 71.52, 71.50, 71.39 (peaks overlapped), 71.36 (peaks overlapped), 71.30 (peaks overlapped), 71.24 (peaks overlapped), 70.5, 67.7, 61.87, 61.80 (peaks overlapped), 61.73, 59.17, 59.06 (peaks overlapped), 59.02, 58.93, 58.3, 57.92, 57.90, 57.84, 57.81, 57.4. HR-MS (ESI-TOF-MS) *m/z*: $[M+Na]^+$ calcd for $C_{138}H_{204}N_2NaO_{60}$ 2873.2905, found 2873.2903.

Compound 8. 7 (185 mg, 64.9 μmol) was dissolved in MeOH (24 mL) and H_2O (12 mL), heated overnight at 50 °C and then heated for 2 h at 60 °C. After the mixture cooled to room temperature, the solvent was removed in vacuum. The residue was dissolved in $CHCl_3$, dried over $MgSO_4$, filtrated, and evaporated in vacuum. The residue was purified by preparative GPC with chloroform as eluent to afford product **8** as a yellow solid (76.3 mg, 41%); ^{1}H-NMR (500 MHz; $CDCl_3$): δ = 8.00 (d, J = 8.3 Hz, 4H, ArH), 7.61 (d, J = 8.3 Hz, 4H, ArH), 7.20 (d, J = 8.8 Hz, 2H, ArH), 6.45-6.43 (m, 4H, ArH), 5.10-2.88 (m, 190H, CD–H, OCH_3, NH_2). ^{13}C-NMR (126 MHz; $CDCl_3$): δ = 163.5, 149.1, 134.2, 132.16 (peaks overlapped), 132.07 (peaks overlapped), 124.4, 121.2, 109.8, 107.8, 105.2,

100.7, 100.4, 100.13, 100.09, 99.9, 98.10, 98.03, 91.4, 90.6, 83.9, 82.65, 82.57 (peaks overlapped), 82.38, 82.22, 82.15, 82.14, 82.02 (peaks overlapped), 81.78, 81.70, 81.43, 81.28, 81.22, 81.19 (peaks overlapped), 81.05, 76.3, 75.2, 72.2, 71.83, 71.67, 71.48, 71.43, 71.22 (peaks overlapped), 71.16, 71.11, 71.02, 70.7, 70.2, 62.08, 62.01 (peaks overlapped), 61.83 (peaks overlapped), 61.77, 59.08, 59.05, 58.93, 58.74 (peaks overlapped), 58.4, 58.04, 57.89, 57.74, 57.67, 57.53. HR-MS (ESI-TOF-MS) *m/z*: $[M+Na]^+$ calcd for $C_{138}H_{204}N_2NaO_{60}$ 2873.2905, found 2873.2898.

2.5 Synthesis of ***unins*-SP-PPE**.

***Unins*-SP-PPE.** To a solution of PPE monomer **7** (75 mg, 26 μmol, 1.0 eq.) in dehydrated acetonitrile (2.6 mL), *tert*-butyl nitrite (62 μL, 0.53 mmol, 20 eq.) and trimethylsilyl azide (103 μL, 0.79 mmol, 30 eq.) was added at 0 °C and then stirred for 2 h at room temperature. After reaction, the solvent was evaporated in vacuum. The residue was diluted with EtOAc and H_2O, and extracted with EtOAc and diethyl ether. The combined organic layer was dried over $MgSO_4$, filtrated and evaporated in vacuum to afford product as a yellow solid. Without further purification, the mixture of the solid, **4** (8.6 mg, 26 μmol, 1.0 eq.), sodium ascorbate (31 mg, 0.16 mmol, 6.0 eq.), and $CuSO_4 \cdot 5H_2O$ (40 mg, 0.16 mmol, 6.0 eq.) in H_2O (659 μL) and ^{t}BuOH (659 μL) was stirred overnight at room temperature. After the reaction, the mixture was diluted with chloroform and saturated NH_4Cl aq., and extracted with chloroform. The combined layer was washed with saturated $NaHCO_3$ aq., dried over $MgSO_4$, filtrated, and evaporated in vacuum. To remove the oligomers, the residue was purified by preparative GPC with chloroform as eluent to afford product ***unins*-SP-PPE** as a yellow solid (33 mg, 39%, $M_w = 4.2 \times 10^4$, $M_n = 3.0 \times 10^4$, PDI = 1.4); ^{1}H-NMR (500 MHz; $CDCl_3$): δ = 8.13 (s, 2H, triazole–H), 7.73-7.46 (m, 14H, ArH), 6.98 (d, 5H, ArH, –CH=CH–), 6.81 (d, J = 8.0 Hz, 1H, ArH), 6.61 (d, J = 6.73 Hz, 1H, ArH), 5.78 (d, J = 9.9 Hz, 1H, –CH=CH–), 5.22-3.01 (m, 186H, CD–H, OCH_3) 2.80 (s, 3H, N–CH_3), 1.40 (s, 3H, *gem*–CH_3), 1.26 (s, 3H, *gem*–CH_3).

2.6 Preparation of films of ***ins*-SP-PPE** and ***unins*-SP-PPE**

Films of ***ins*-SP-PPE** and ***unins*-SP-PPE** were prepared by drop-casting 30 μL of $CHCl_3$-CH_3CN (1:9) solution of the polymer (1×10^{-4} M for the SP units) onto glass plates (1 cm×1 cm). After the drop-casting, the films were dried at room temperature.

3. NMR spectra

3.1 NMR spectra of **S1**

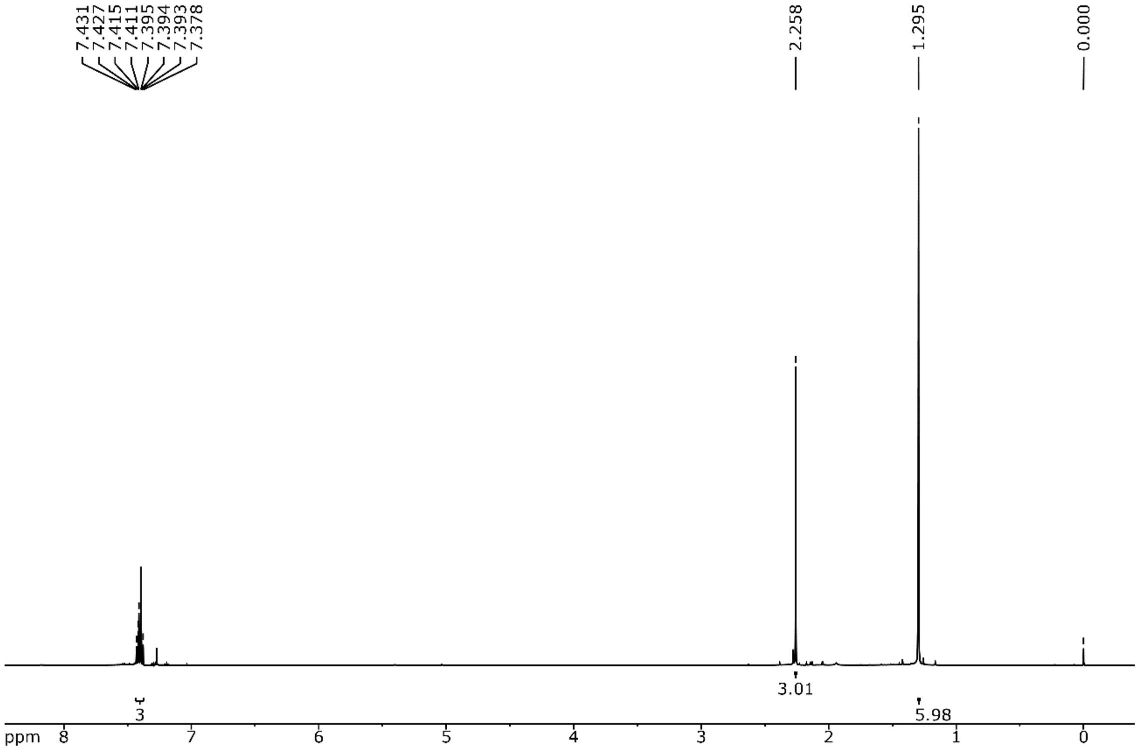

Figure S1. ^{1}H-NMR spectrum of **S1** (500 MHz, $CDCl_3$).

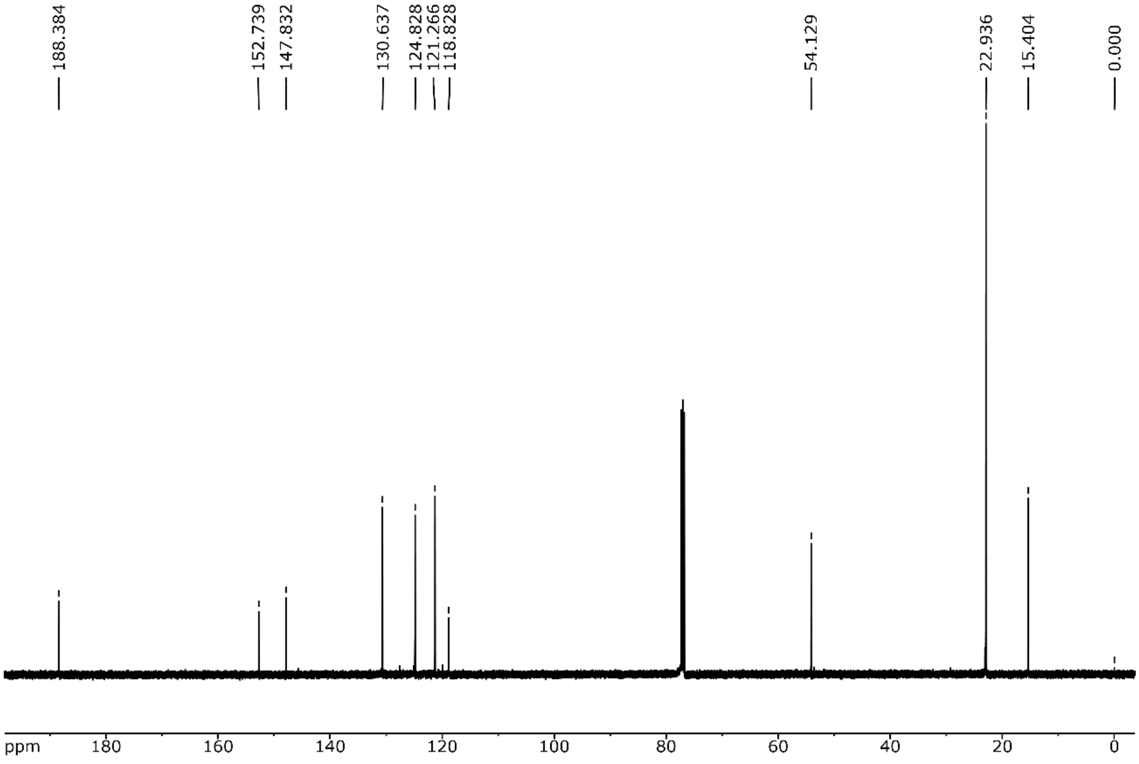

Figure S2. ^{13}C-NMR spectrum of **S1** (126 MHz, $CDCl_3$).

3.2 NMR spectra of **S2**

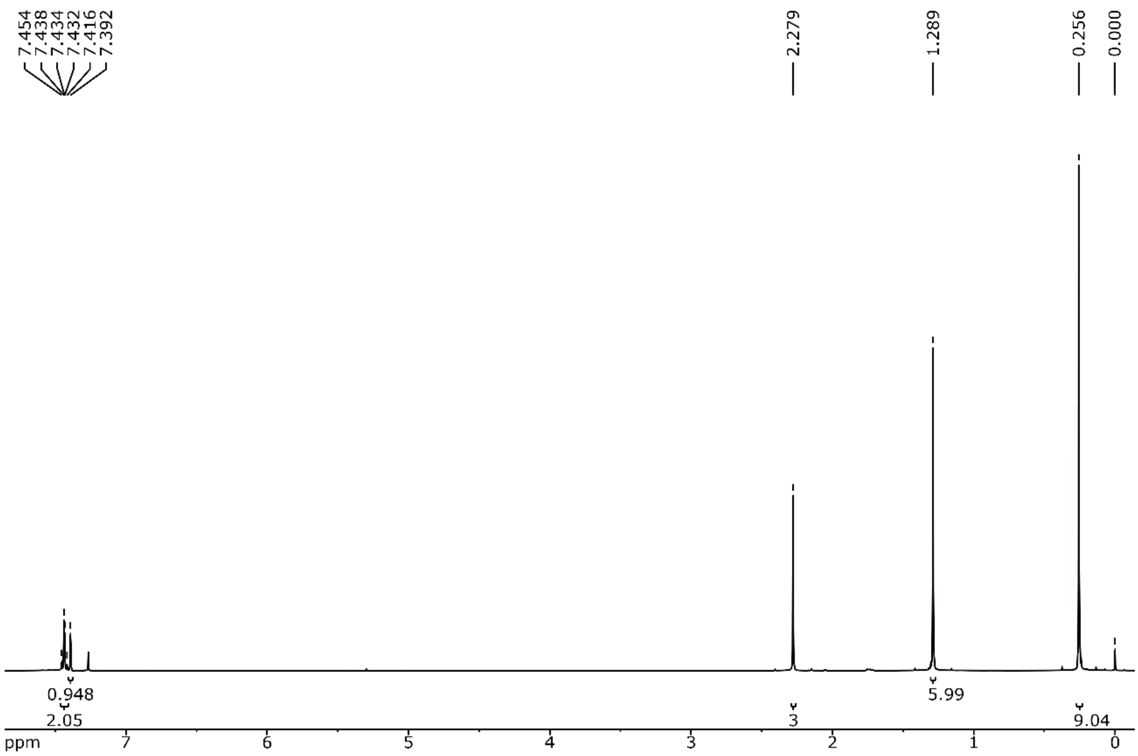

Figure S3. ^{1}H-NMR spectrum of **S2** (500 MHz, $CDCl_3$).

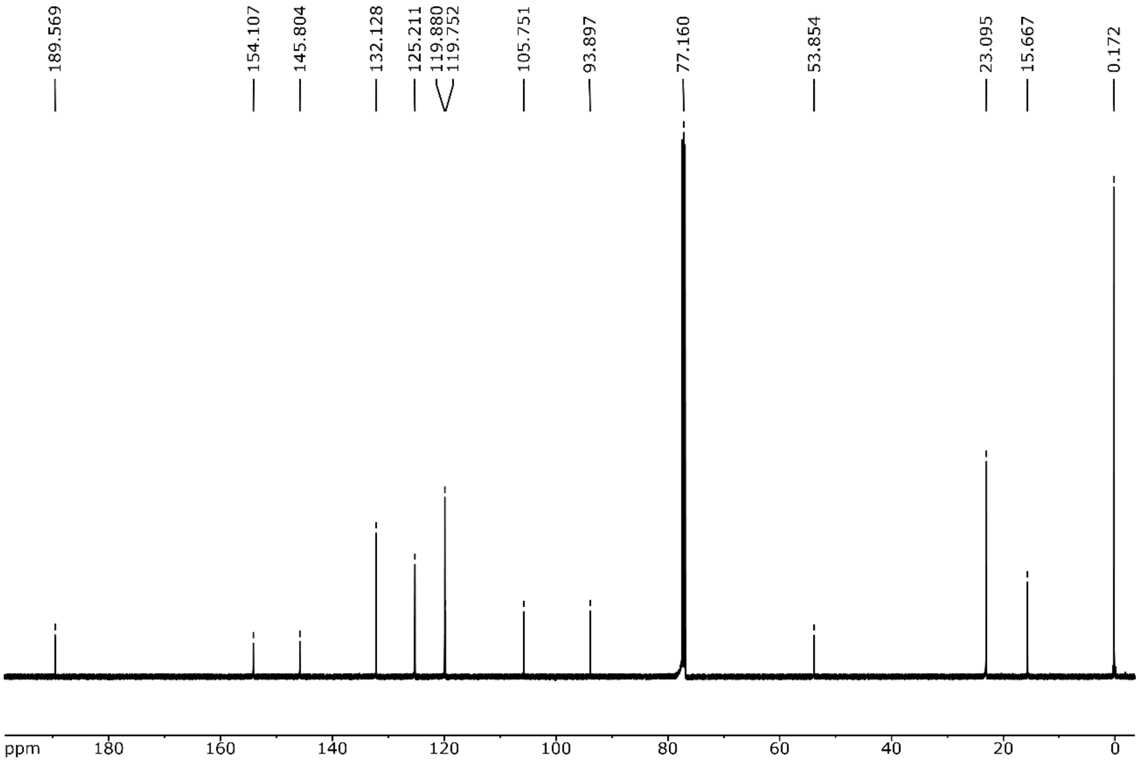

Figure S4. ^{13}C-NMR spectrum of **S2** (126 MHz, $CDCl_3$).

3.3 NMR spectrum of **2**

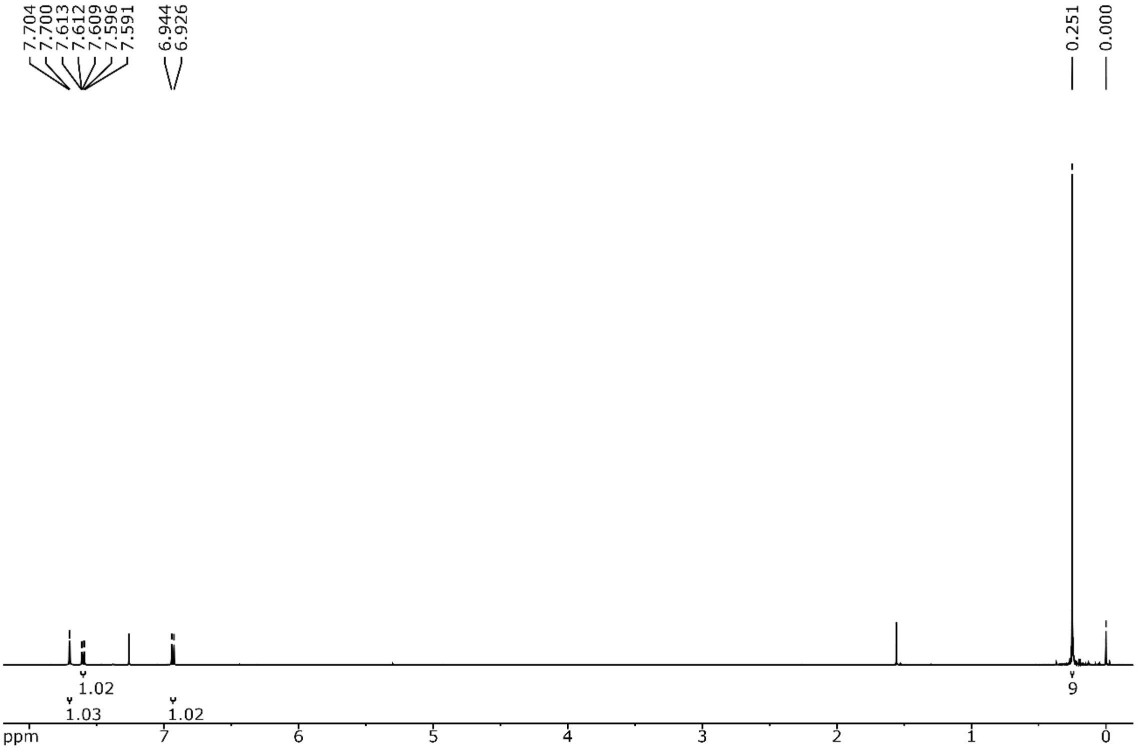

Figure S5. ^{1}H-NMR spectrum of **2** (500 MHz, $CDCl_3$).

3.4 NMR spectra of **3**

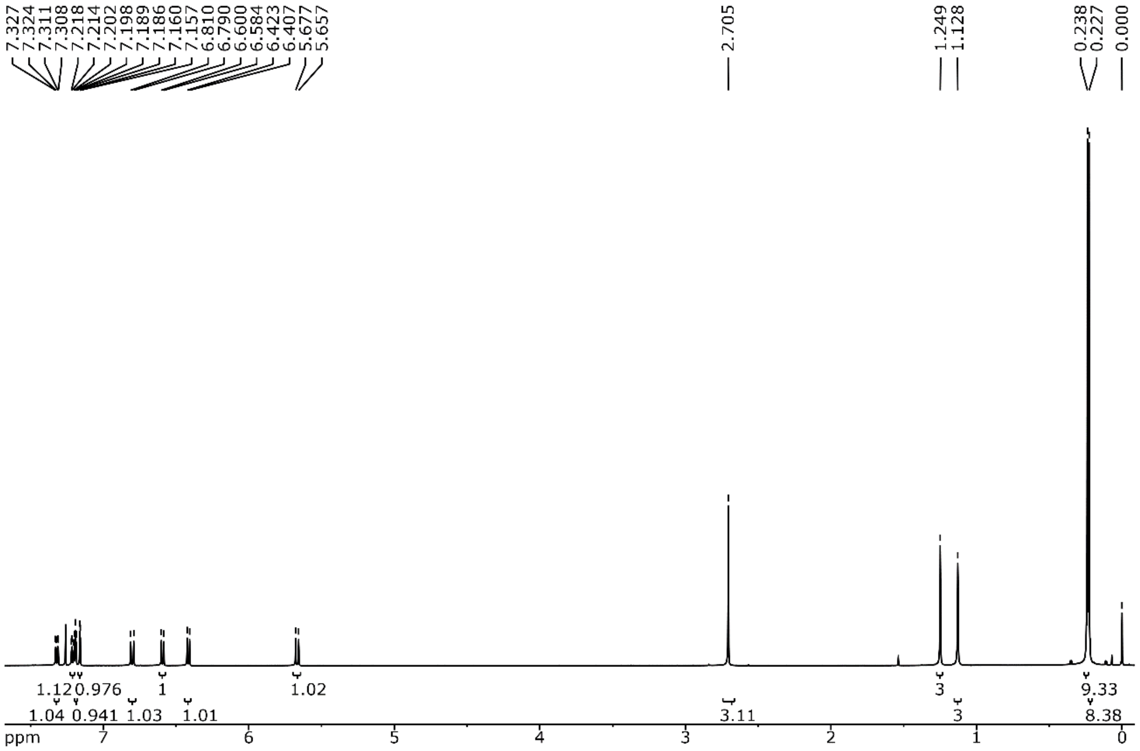

Figure S6. ^{1}H-NMR spectrum of **3** (500 MHz, $CDCl_3$).

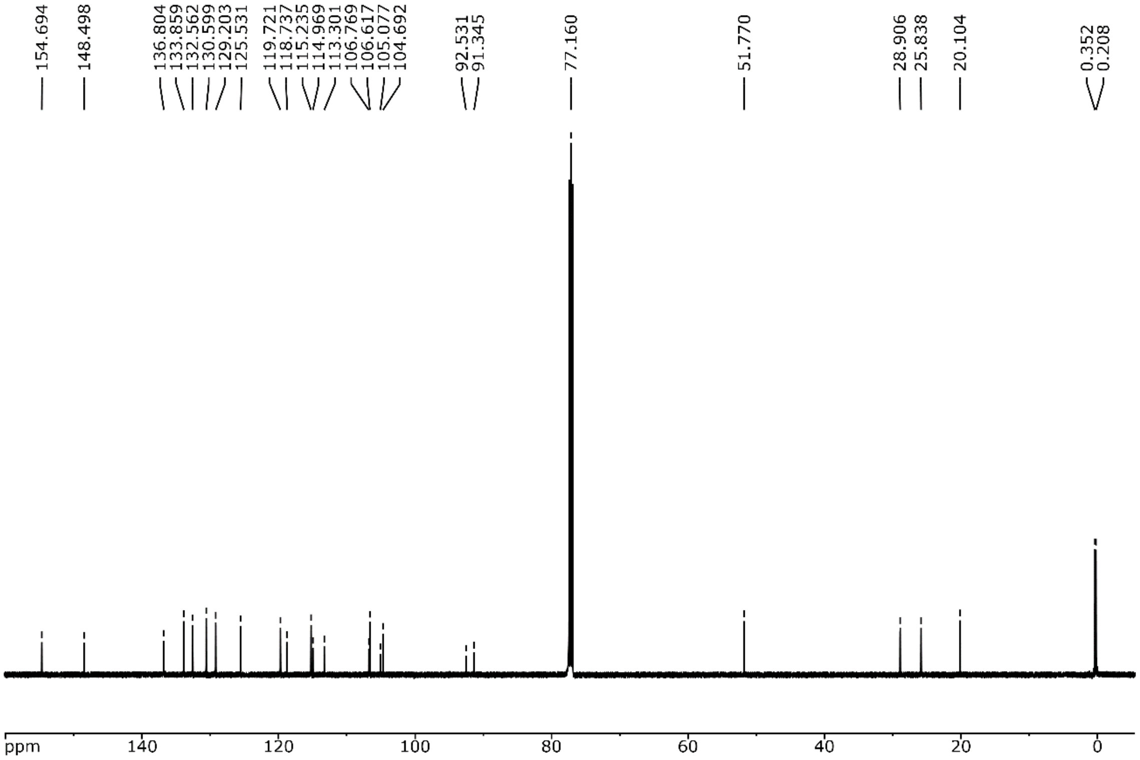

Figure S7. ^{13}C-NMR spectrum of **3** (126 MHz, $CDCl_3$).

3.5 NMR spectra of **4**

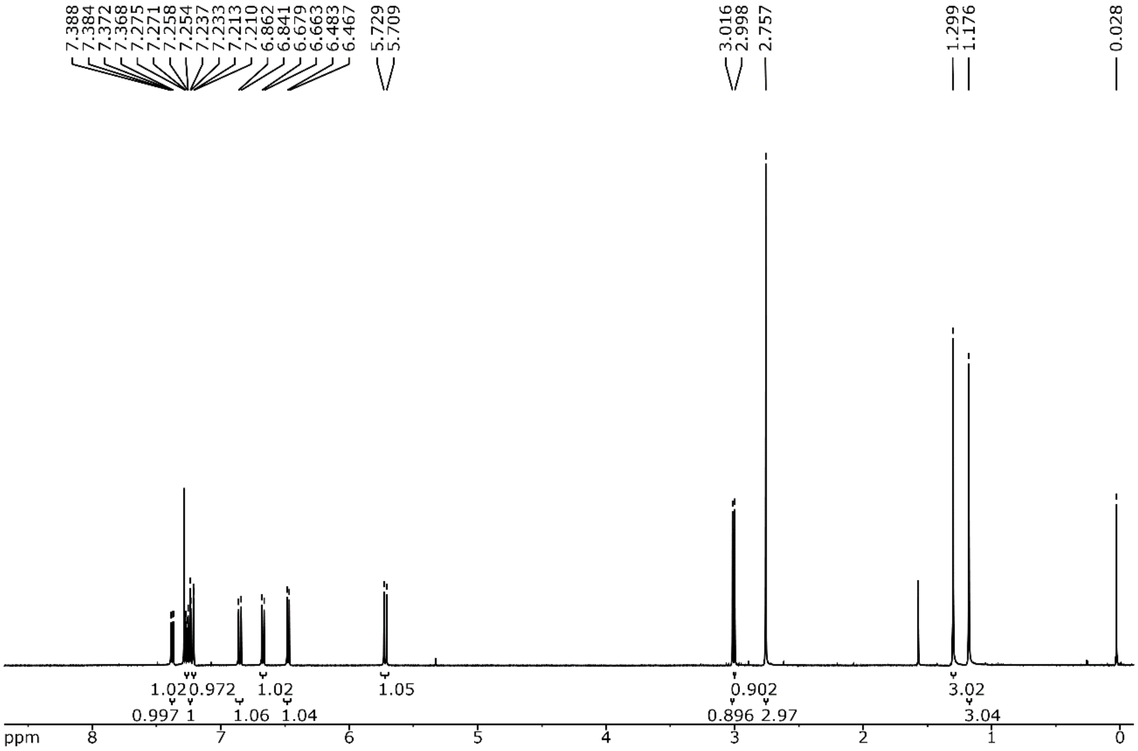

Figure S8. ^{1}H-NMR spectrum of **4** (500 MHz, $CDCl_3$).

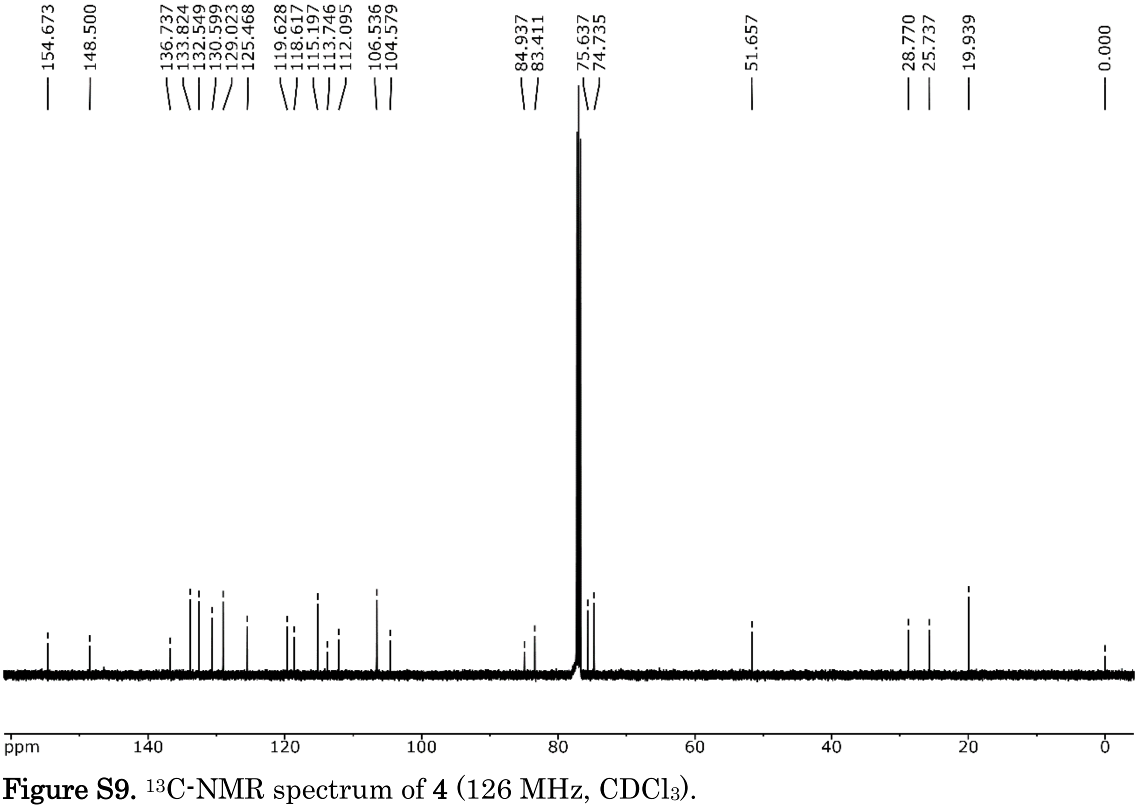

Figure S9. ^{13}C-NMR spectrum of **4** (126 MHz, $CDCl_3$).

3.6 NMR spectra of **7**

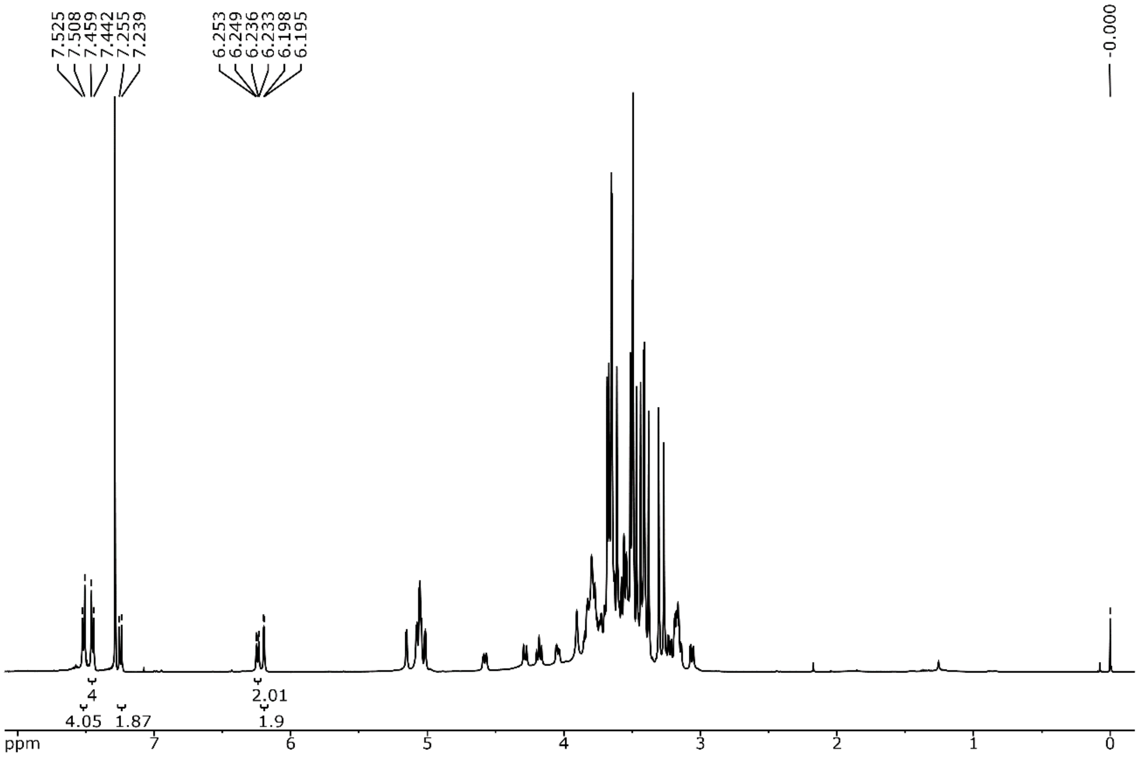

Figure S10. ^{1}H-NMR spectrum of **7** (500 MHz, $CDCl_3$).

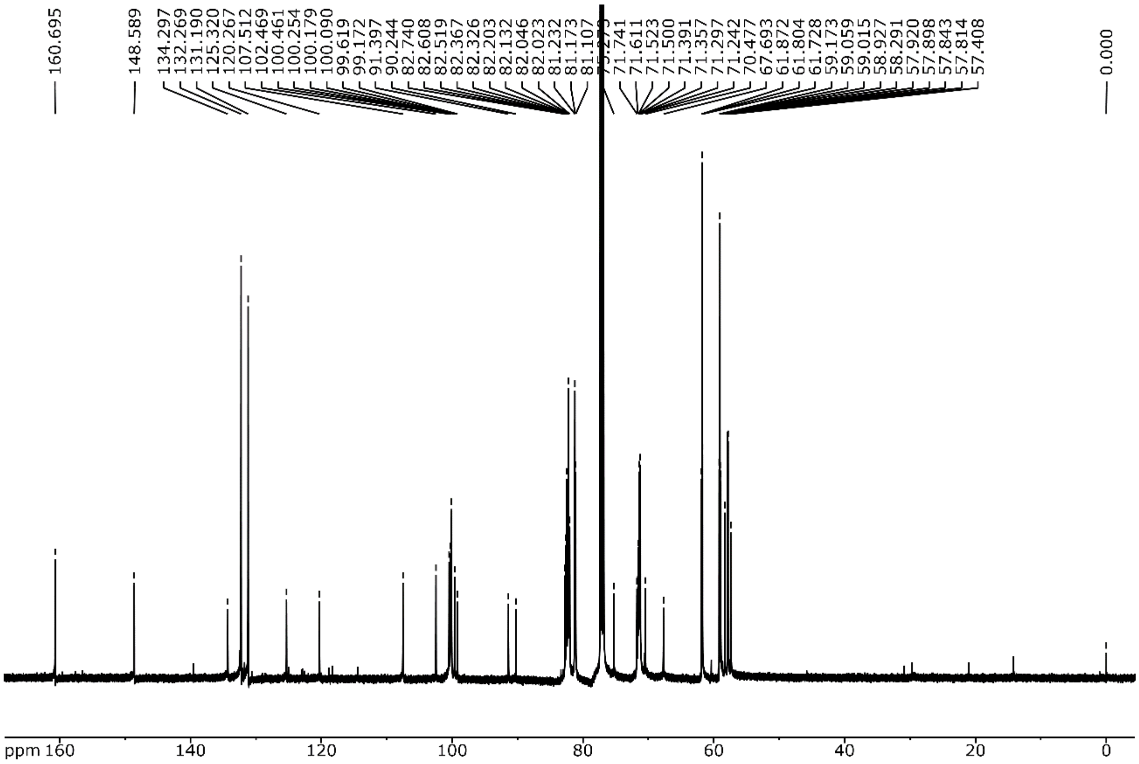

Figure S11. ^{13}C-NMR spectrum of **7** (126 MHz, $CDCl_3$).

3.7 NMR spectra of **8**

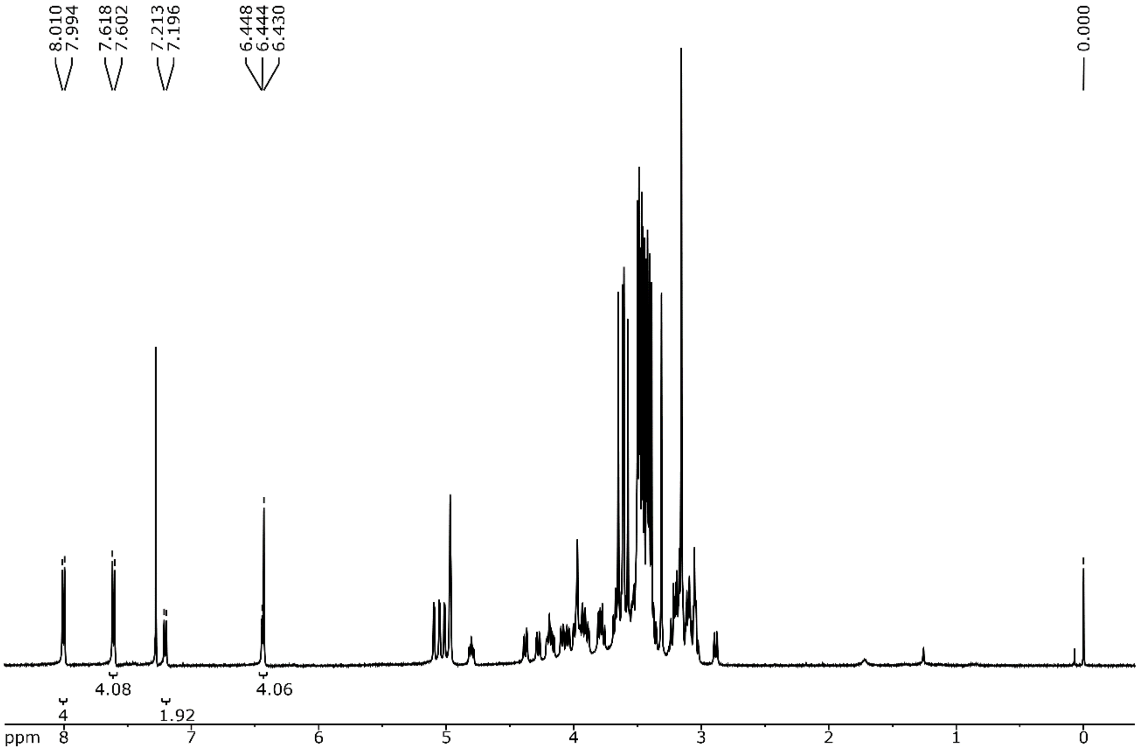

Figure S12. ^{1}H-NMR spectrum of **8** (500 MHz, $CDCl_3$).

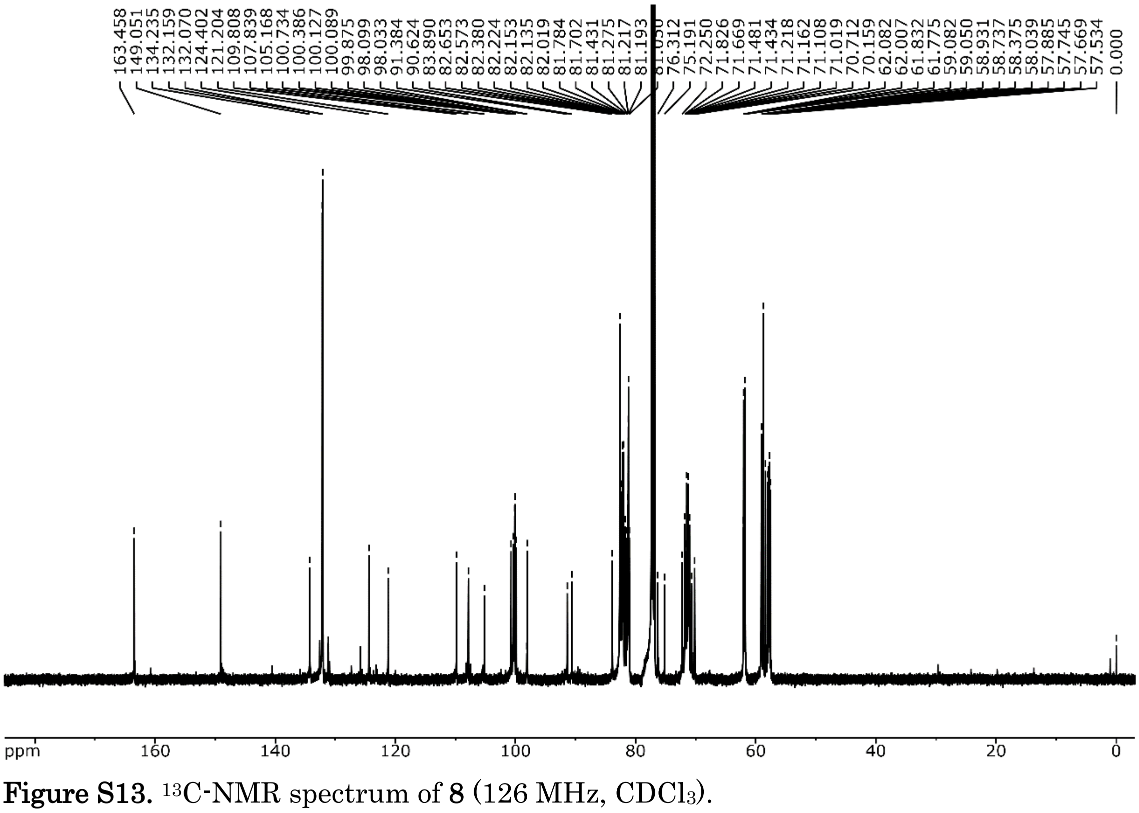

Figure S13. ^{13}C-NMR spectrum of **8** (126 MHz, $CDCl_3$).

3.8 NMR spectra of 7 and 8 (aromatic region)

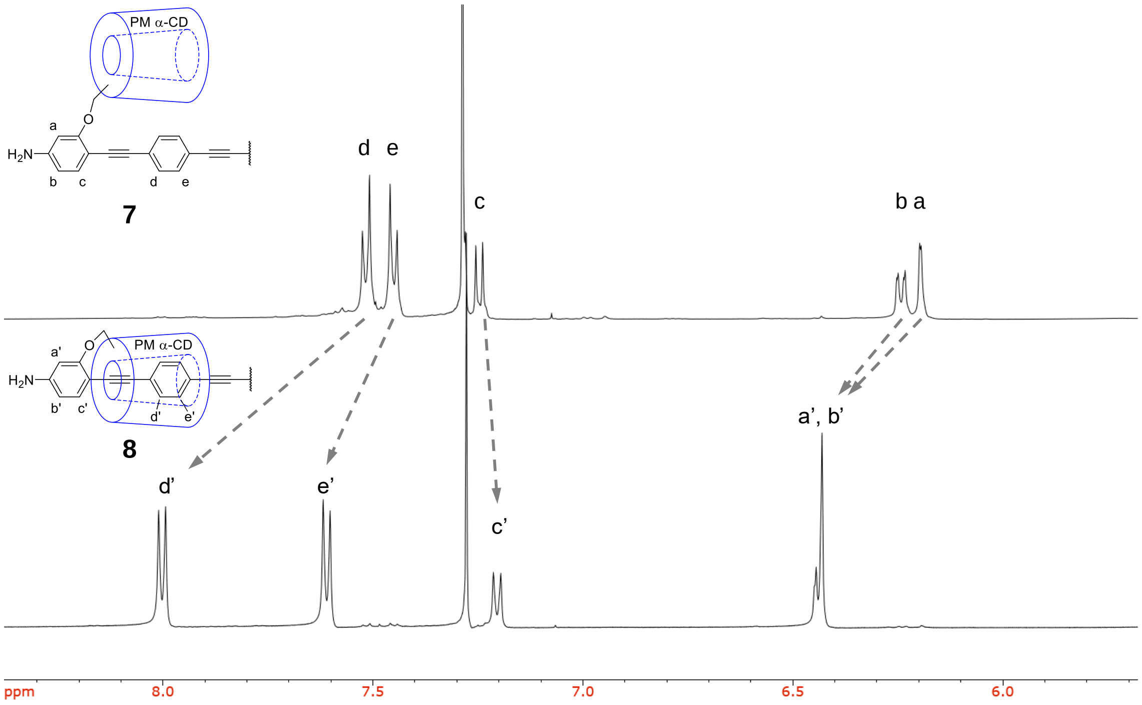

Figure S14. ^{1}H-NMR spectrum (aromatic region) of **7** and **8** (500 MHz, $CDCl_3$).

3.9 NMR spectra of ***ins*-SP-PPE** and ***unins*-SP-PPE**

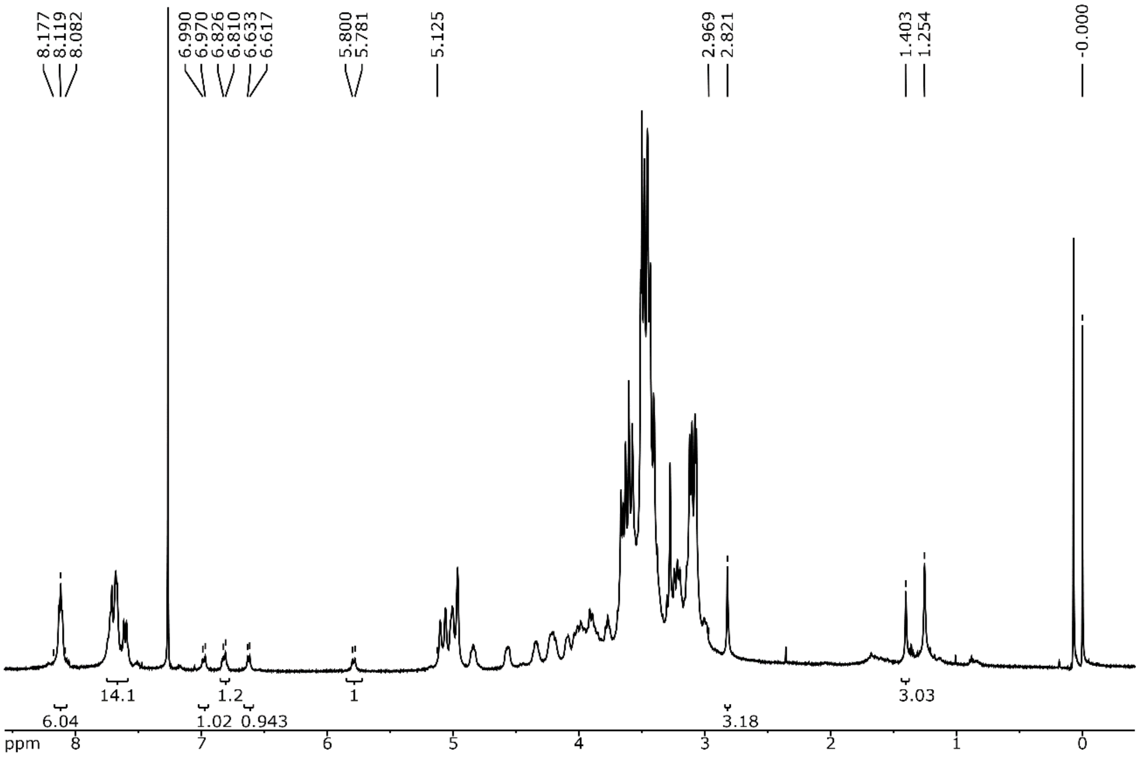

Figure S15. ^{1}H-NMR spectrum of ***ins*-SP-PPE** (500 MHz, $CDCl_3$).

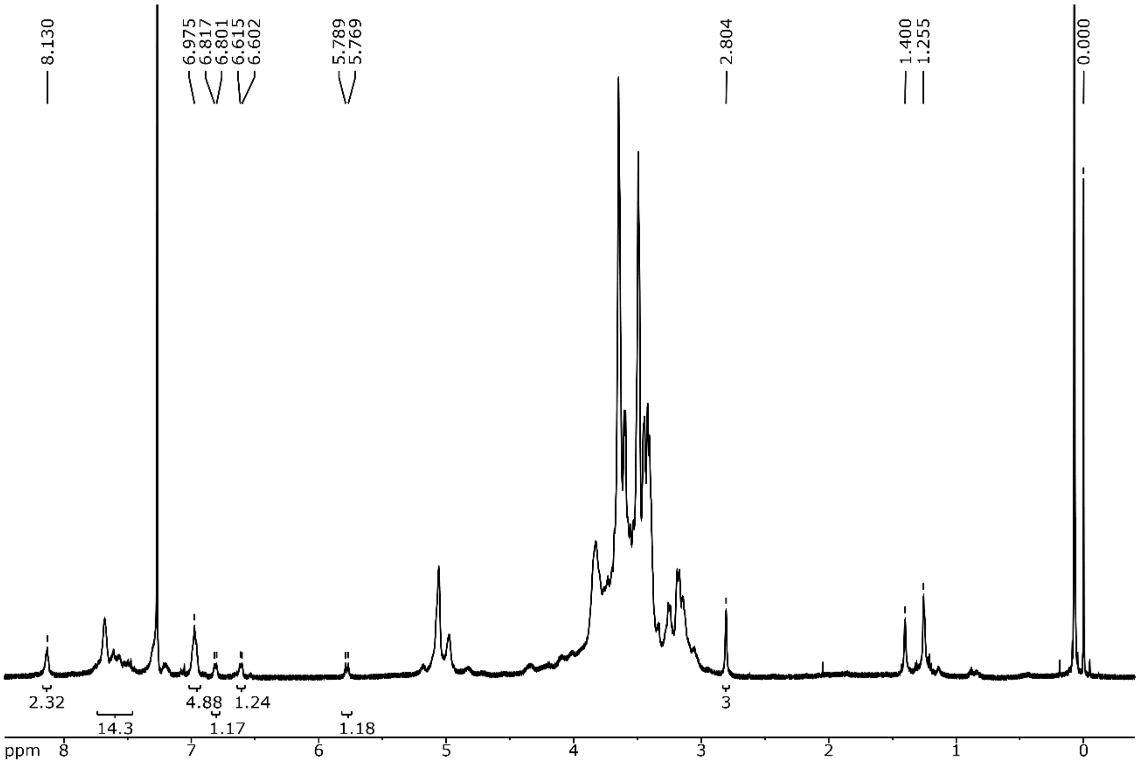

Figure S16. ^{1}H-NMR spectrum of ***unins*-SP-PPE** (500 MHz, $CDCl_3$).

3.10 NMR titration

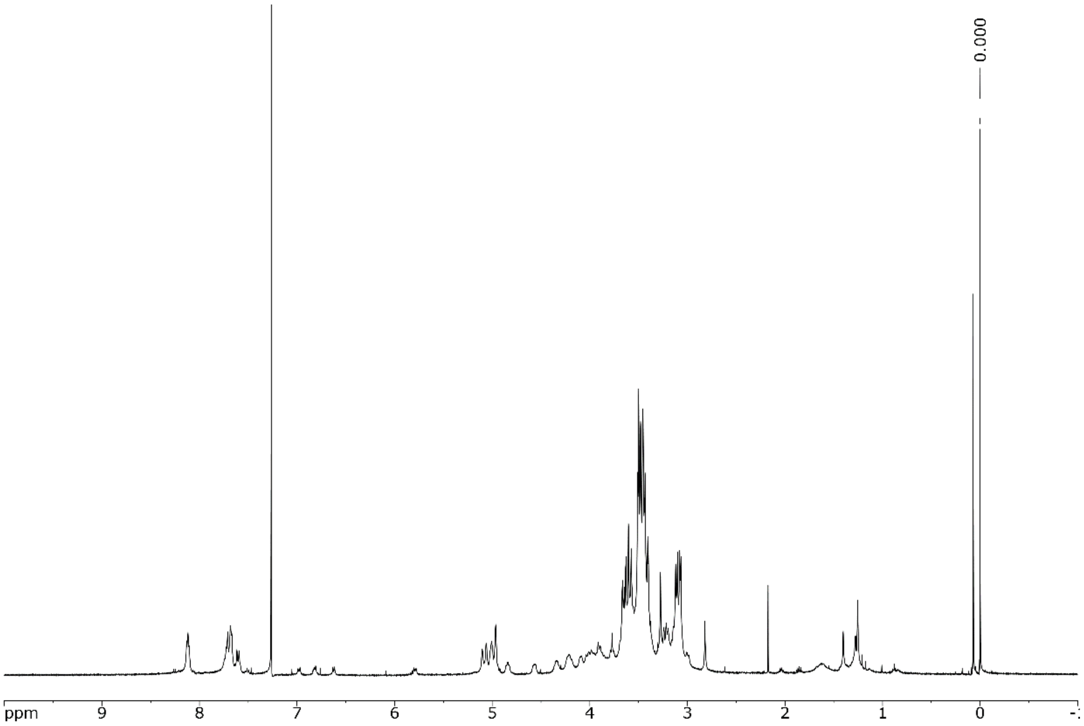

Figure S17. ^{1}H-NMR spectrum of ***ins*-SP-PPE** (500 MHz, $CDCl_3$) before the addition of an acid.

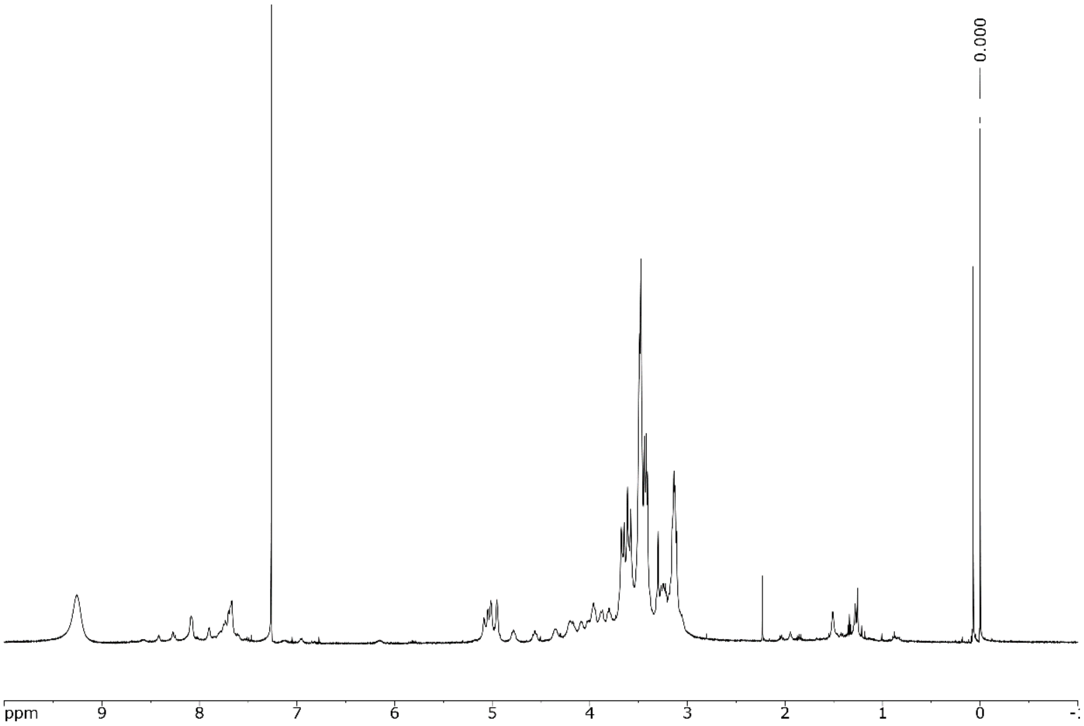

Figure S18. ^{1}H-NMR spectrum of ***ins*-SP-PPE** (500 MHz, $CDCl_3$) after adding TFA (0.04 M).

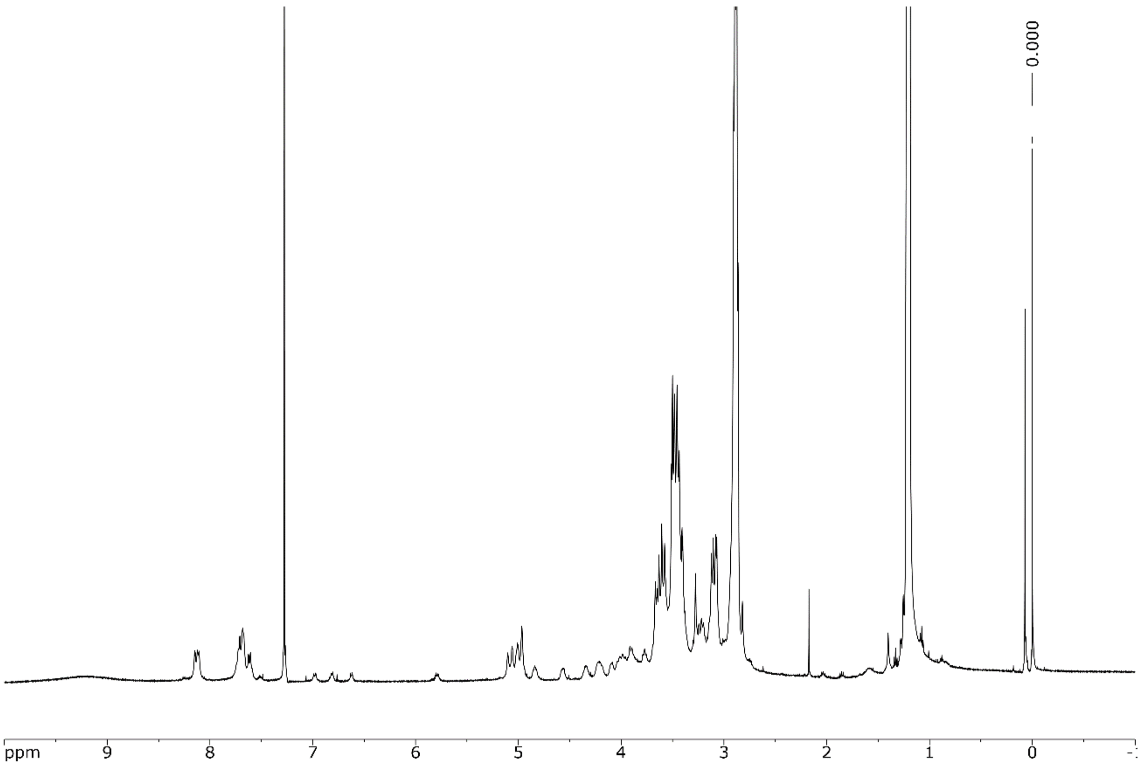

Figure S19. ^{1}H-NMR spectrum of ***ins*-SP-PPE** (500 MHz, $CDCl_3$) after adding TFA (0.04 M) and then TEA (0.08 M).

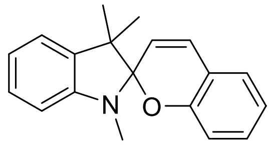

Scheme S5. A chemical structure of unsubstituted SP.

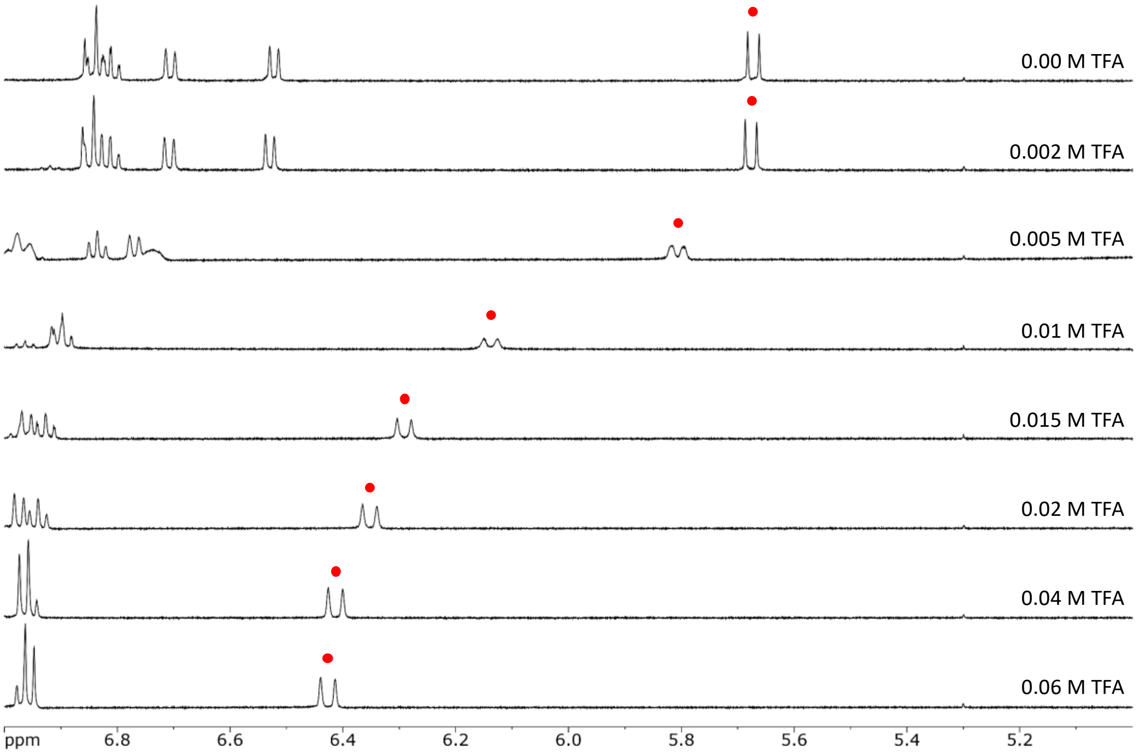

Figure S20. ^{1}H-NMR spectra (aromatic region) of unsubstituted SP upon addition of different amounts of TFA (0 – 0.06 M). Red dots indicate signals deriving from vinyl protons of SP and MCH^{+}.

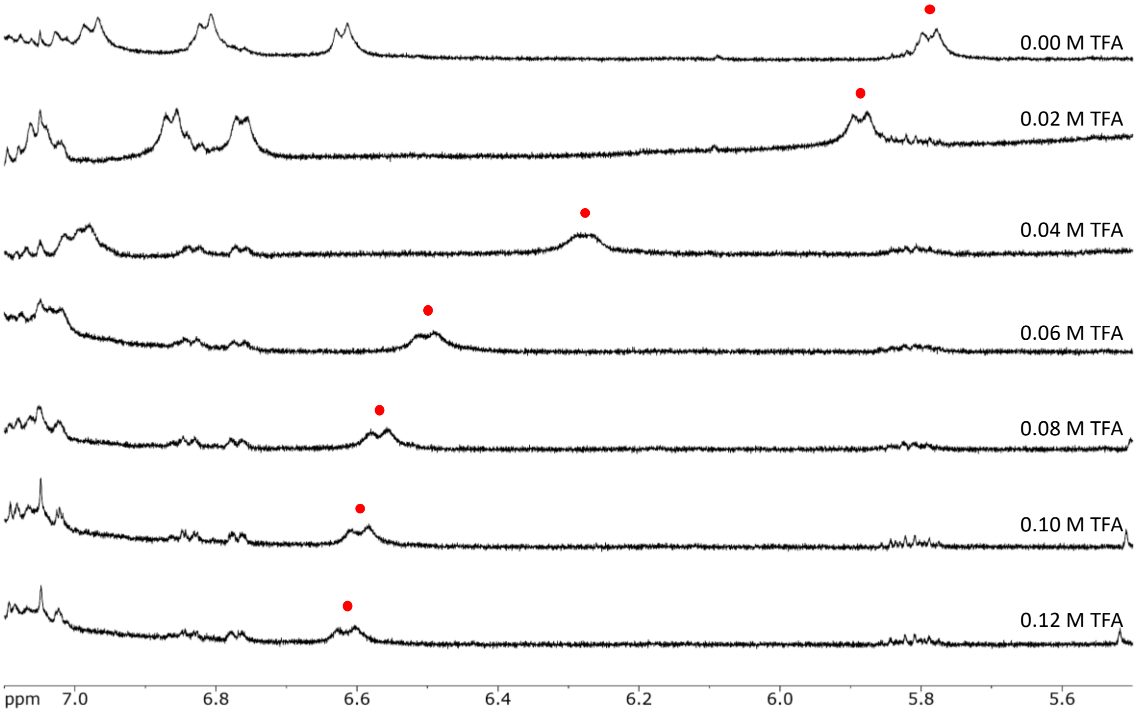

Figure S21. ^{1}H-NMR spectra (aromatic region) of ***ins*-SP-PPE** upon addition of different amounts of TFA (0 – 0.12 M). Red dots indicate signals deriving from vinyl protons of SP and MCH^+.

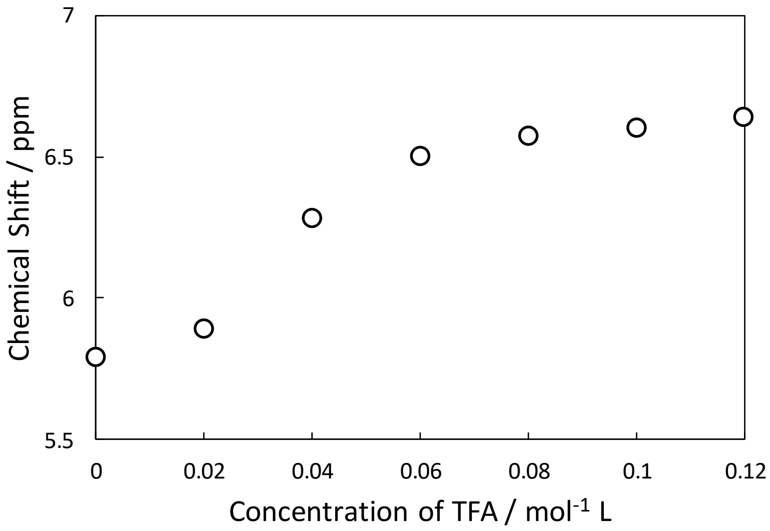

Figure S22. A chemical shift change of the signals of vinyl protons of SP and MCH^+ in ***ins*-SP-PPE** under the addition of TFA (0 – 0.12 M).

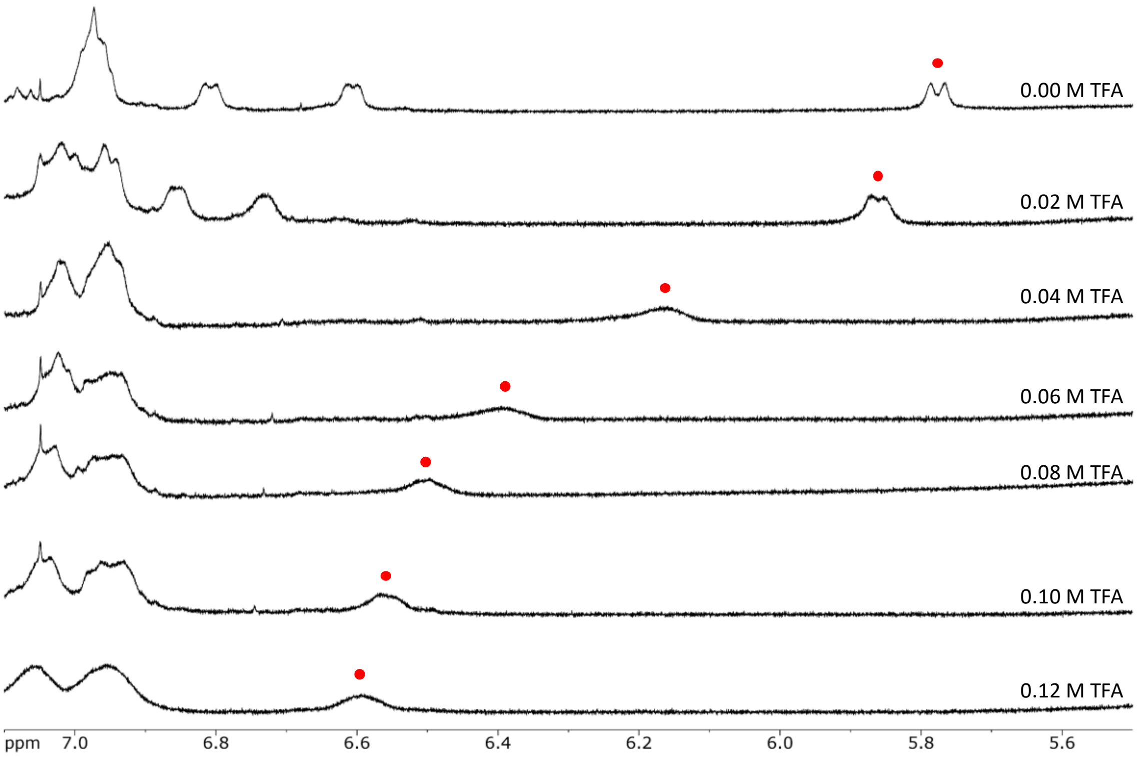

Figure S23. ^{1}H-NMR spectra (aromatic region) of ***unins*-SP-PPE** upon addition of different amounts of TFA (0 – 0.12 M). Red dots indicate signals deriving from a vinyl proton of SP and MCH^+.

4. GPC charts

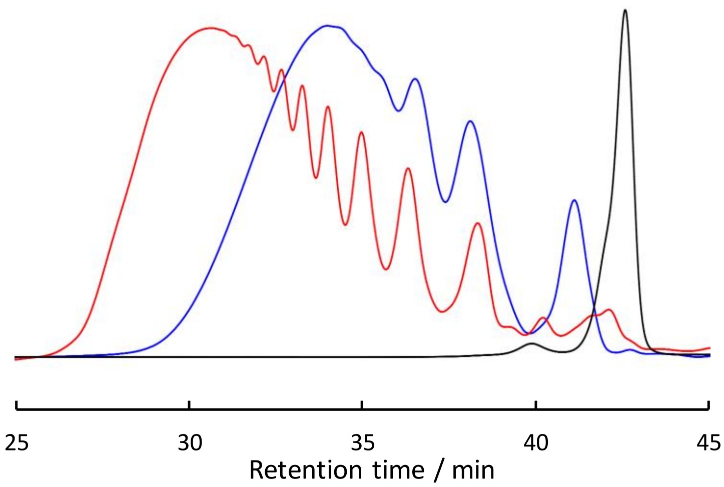

Figure S24. GPC analysis of monomer **8** (gray line), crude ***ins*-SP-PPE** (red line) and crude ***unins*-SP-PPE** (blue line) after polymerization.

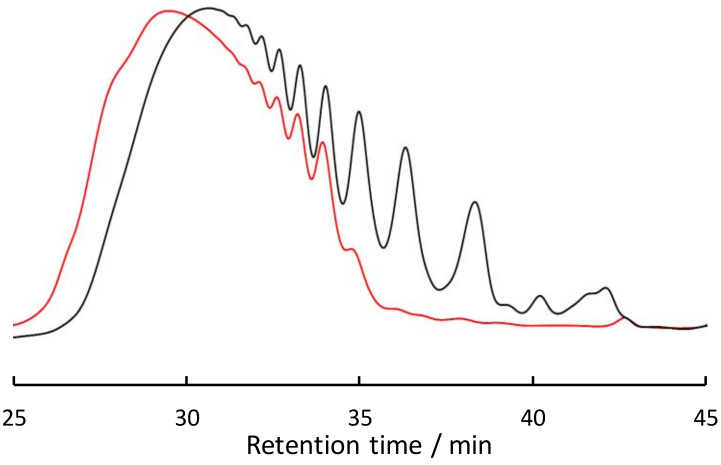

Figure S25. GPC analysis of crude (black line) and purified (red line) *ins*-SP-PPE.

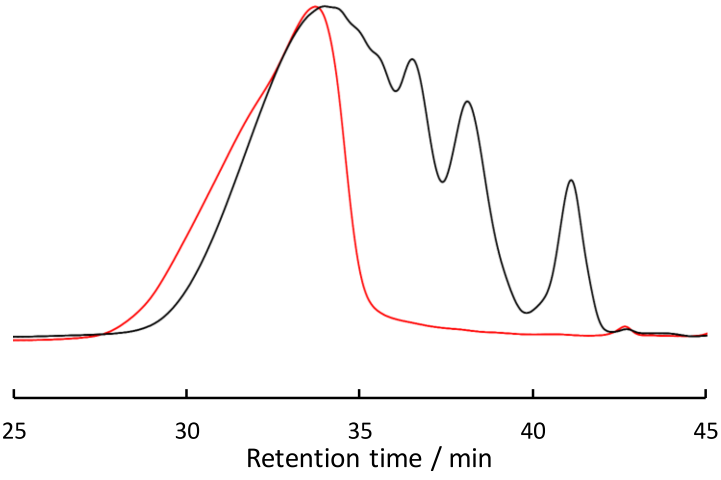

Figure S26. GPC analysis of crude (black line) and purified (red line) *unins*-SP-PPE.

5. UV-Vis spectra

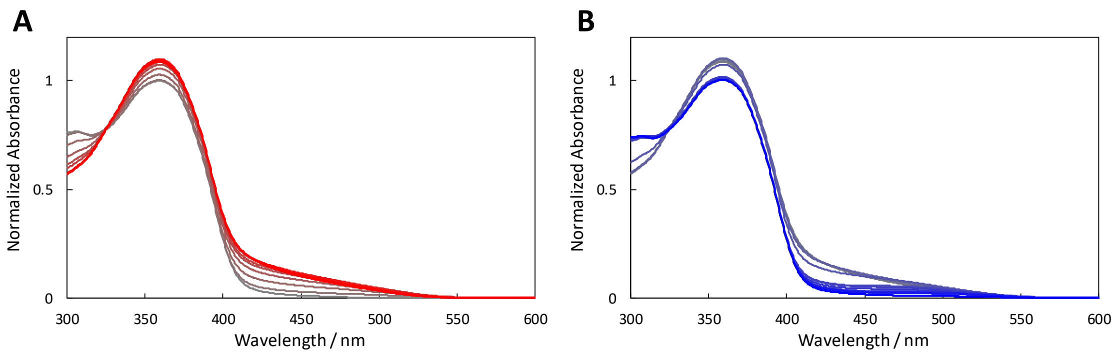

Figure S27. UV-Vis spectra of ***unins*-SP-PPE** (2.0 × 10^{-5} M in CH_2Cl_2) upon addition of different amounts of (A) TFA (0 – 0.2 M) and then (B) TEA (0 – 0.2 M).

6. Photoluminescence study

The acid-induced quenching of the emission of ***ins*-SP-PPE** was observed in the solution and solid states. In the solution, the quenching was not completely reversible (Figure S23), which may be caused by an incomplete conversion of MCH^+ to SPs or partial photochromic isomerization of SPs to MCs. The quenching was also observed in ***unins*-SP-PPE** and was irreversible (Figure S24). The emission peak was blue-shifted after the addition of an acid and a base, which indicated the low durability of ***unins*-SP-PPE**. In the solid state, the quenched emission of the film was not able to completely recover by dipping the film to NH_3 aqueous solution (Figure S25), which is consistent with the result in the solution.

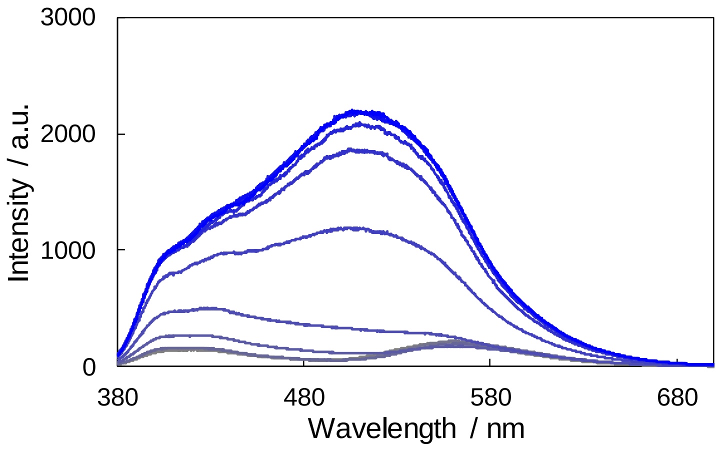

Figure S28. Emission spectra of ***ins*-SP-PPE** (2.0 × 10^{-5} M in CH_2Cl_2) upon adding different amount of TEA (0 – 0.2 M) after the addition of TFA (0.2 M).

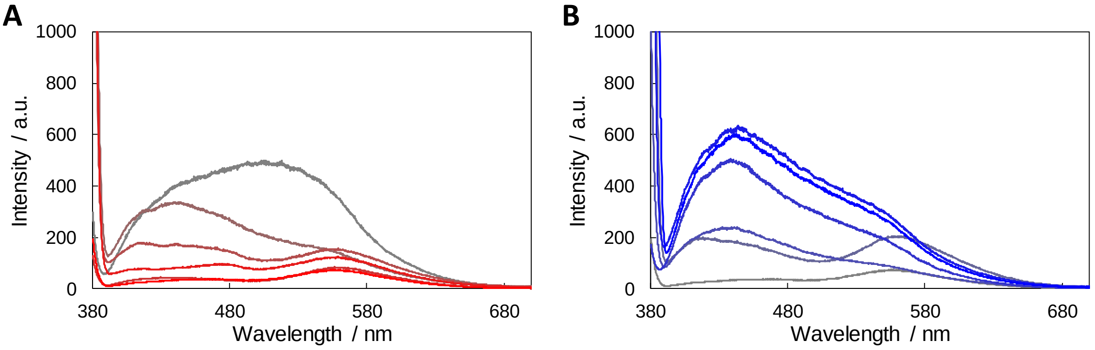

Figure S29. Emission spectra of ***unins*-SP-PPE** (2.0×10^{-5} M in CH_2Cl_2) upon adding different amount of (A) TFA (0 – 0.2 M) and then (B) TEA (0 – 0.2 M).

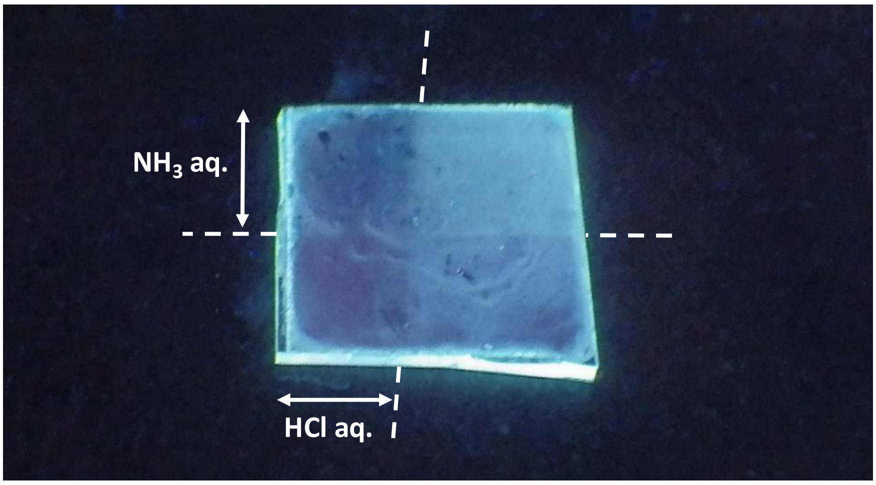

Figure S30. Fluorescence quenching and partial recovery

5. References

1. Terao, J.; Wadahama, A.; Fujihara, T.; Tsuji, Y. Synthesis of linked symmetric [3]rotaxane having an oligomeric phenylene–ethynylene unit as a π guest via double sonogashira cross-coupling. *Chem. Lett.* **2010**, *39*, 518–519.
2. Balmond, E. I.; Tautges, B. K.; Faulkner, A. L.; Or, V. W.; Hodur, B. M.; Shaw, J. T.; Louie, A. Y. Comparative Evaluation of Substituent Effect on the Photochromic Properties of Spiropyrans and Spirooxazines. *J. Org. Chem.* **2016**, *81*, 8744–8758.
3. Wu, I.-C.; Yu, J.; Ye, F.; Rong, Y.; Gallina, M.E.; Fujimoto, B.S.; Zhang, Y.; Chan, Y.-H.; Sun, W.; Zhou, X.-H.; Wu, C.; Chiu, D.T. Squaraine-Based Polymer Dots with Narrow, Bright Near-Infrared Fluorescence for Biological Applications. *J. Am. Chem. Soc.* **2014**, *137*, 173–178.
4. Walkey, M.C.; Byrne, L.T.; Piggott, M.J.; Low, P.J.; Koutsantonis, G.A. Enhanced bi-stability in a ruthenium alkynyl spiropyran complex. *Dalton Trans.* **2015**, *44*,

8812–8815.

5. Chang, K.-H.; Huang, C.-C.; Liu, Y.-H.; Hu, Y.-H.; Chou, P.-T.; Lin, Y.-C. Synthesis of photo-luminescent Zn(II) Schiff base complexes and its derivative containing Pd(II) moiety. *Dalton Trans.* **2004**, 1731–1738.
